# Optimizing the Procedure to Manufacture Clinical-Grade NK Cells for Adoptive Immunotherapy

**DOI:** 10.3390/cancers13030577

**Published:** 2021-02-02

**Authors:** Adrián Fernández, Alfonso Navarro-Zapata, Adela Escudero, Nerea Matamala, Beatriz Ruz-Caracuel, Isabel Mirones, Alicia Pernas, Marta Cobo, Gema Casado, Diego Lanzarot, Carlos Rodríguez-Antolín, María Vela, Cristina Ferreras, Carmen Mestre, Aurora Viejo, Alejandra Leivas, Joaquín Martínez, Lucía Fernández, Antonio Pérez-Martínez

**Affiliations:** 1 Hematological Malignancies Lab-H12O Clinical Research Unit, Spanish National Cancer Research Centre (CNIO), 28029 Madrid, Spain; afmartin@cnio.es (A.F.); aleivas@ext.cnio.es (A.L.); jmarti01@med.ucm.es (J.M.); lvfernandez@cnio.es (L.F.); 2 Translational Research Group in Paediatric Oncology Haematopoietic Transplantation & Cell Therapy, La Paz University Hospital Institute for Health Research-IdiPAZ, 28046 Madrid, Spain; alfonso.navarro.zapata@idipaz.es (A.N.-Z.); maria.vela@idipaz.es (M.V.); cristina.ferreras@salud.madrid.org (C.F.); carmen.mestre.duran@idipaz.es (C.M.); 3 Institute of Medical and Molecular Genetics (INGEMM), La Paz University Hospital, 28046 Madrid, Spain; adela.escudero@idipaz.es; 4 Translational Research in Pediatric Oncology, Hematopoietic Transplantation & Cell Therapy, La Paz University Hospital Institute for Health Research-Institute of Medical and Molecular Genetics (INGEMM-IdiPAZ), 28046 Madrid, Spain; nerea.matamala.zamarro@idipaz.es (N.M.); beatriz.ruz@salud.madrid.org (B.R.-C.); 5 Advanced Therapy Medicinal Products Production Unit Pediatric Hemato-Oncology Department, La Paz University Hospital, 28046 Madrid, Spain; isabel.mirones@salud.madrid.org (I.M.); alicia.pernas@salud.madrid.org (A.P.); marta.cobo@salud.madrid.org (M.C.); mgema.casado@salud.madrid.org (G.C.); 6 Advanced Therapy Medicinal Products Production Unit, Pediatric Hemato-Oncology Service and Pharmacy Service, La Paz University Hospital, 28046 Madrid, Spain; 7 Applications Department Miltenyi Biotec, 28223 Madrid, Spain; diego@miltenyi.com; 8 Experimental Therapies and Novel Biomarkers in Cancer, La Paz University Hospital Institute for Health Research-IdiPAZ, 28046 Madrid, Spain; crodrigueza@salud.madrid.org; 9 Hematology and Hemotherapy Department, La Paz University Hospital, 28046 Madrid, Spain; aurora.viejo@salud.madrid.org; 10 Hematology Department 12 de Octubre University Hospital, 28041 Madrid, Spain; 11 Pediatric Hemato-Oncology Department, La Paz University Hospital, 28046 Madrid, Spain

**Keywords:** NK cell immunotherapy, NK cell activation and expansion, NKAE cells, clinical-grade manufacturing, CliniMACS Prodigy

## Abstract

**Simple Summary:**

Natural Killer cells have shown promise to treat different malignancies. Several methods have been described to obtain fully activated NK cells for clinical use. Here, we use different cell culture media and different artificial antigen presenting cells to optimize a GMP compliant manufacturing method to obtain activated and expanded NK cells suitable for clinical use.

**Abstract:**

Natural killer (NK) cells represent promising tools for cancer immunotherapy. We report the optimization of an NK cell activation–expansion process and its validation on clinical-scale. Methods: RPMI-1640, stem cell growth medium (SCGM), NK MACS and TexMACS were used as culture mediums. Activated and expanded NK cells (NKAE) were obtained by coculturing total peripheral blood mononuclear cells (PBMC) or CD45RA^+^ cells with irradiated K562mbIL15-41BBL or K562mbIL21-41BBL. Fold increase, NK cell purity, activation status, cytotoxicity and transcriptome profile were analyzed. Clinical-grade NKAE cells were manufactured in CliniMACS Prodigy. Results: NK MACS and TexMACs achieved the highest NK cell purity and lowest T cell contamination. Obtaining NKAE cells from CD45RA^+^ cells was feasible although PBMC yielded higher total cell numbers and NK cell purity than CD45RA^+^ cells. The highest fold expansion and NK purity were achieved by using PBMC and K562mbIL21-41BBL cells. However, no differences in activation and cytotoxicity were found when using either NK cell source or activating cell line. Transcriptome profile showed to be different between basal NK cells and NKAE cells expanded with K562mbIL21-41BBL or K562mbIL15-41BBL. Clinical-grade manufactured NKAE cells complied with the specifications from the Spanish Regulatory Agency. Conclusions: GMP-grade NK cells for clinical use can be obtained by using different starting cells and aAPC.

## 1. Introduction

Development of new donor lymphocyte infusions products with antitumor reactivity and reduced graft-versus-host disease (GvHD) risk represents a challenging issue in cancer immunotherapy. Natural killer (NK) cells are lymphocytes from the innate immunity with the ability to recognize and target tumor cells without prior sensitization, making them ideal therapeutic agents to treat cancer [1]. In fact, NK cell infusions are well tolerated, do not cause GvHD or autoimmunity and have been associated with complete remission in poor-prognosis patients with acute myeloid leukemia (AML) [2,3]. One limitation of using NK cells as a therapeutic tool is that they have to be fully activated and have to be infused in large numbers to observe a clinical benefit. However, activation status and NK cell numbers are low in the main sources of NK cells: PBMCs or umbilical cord blood units. To overcome this limitation, different protocols have been designed to expand and activate NK cells ex vivo before transfer to the patient. They may differ in the addition of different cytokines [4,5,6,7], the source of NK cells used [8,9], or the coculture with distinct irradiated feeder or artificial antigen presenting cells (aAPC) [10,11,12], among others. Nevertheless, ex vivo activated and expanded NK cells (NKAE) share some common features [13]. In general, ex vivo NKAE cells show an increased activation status and increased cytotoxicity, even responding against tumor targets apparently resistant to NK cell lysis [10,14,15].

Several groups have developed aAPC by engineering the K562 cell line to express the membrane-bound form of interleukin (IL)-15 (mIL-15) or IL21 and CD137 ligand. The combination of activating signals provided by the K562 cell line, costimulation via 4-1BBL (CD137L) and survival signals provided by cytokines such as IL15 or IL21 can mediate NK cell proliferation and the expansion of highly cytotoxic NK cells [11,12].

Besides, the transfer of these protocols to the clinical scale in a manageable, good manufacturing practice (GMP)-compliant way is still challenging. The multitude of necessary hands-on steps complicates the routine use of these scaled-up manual approaches as standard therapy. Recently, Miltenyi Biotec has allowed the automated clinical-scale manufacturing of NK cells using centrifugation, magnetic cell separation and cell cultivation within a closed system, namely the CliniMACS Prodigy [16]. By using this device, different groups have designed and optimized successful NK cell expansion protocols [17,18,19]. 

In parallel, improvements are also in development concerning T-cell depletion of the allogeneic hematopoietic stem cell transplantation (HSCT) graft, as it may lead to increased graft failure, relapse and infections due to delayed immune recovery. The selective depletion of the CD45RA^+^ subset can reduce GvHD through removal of naïve T cells [20]. This fraction, usually discarded, contains NK cells and potentially could be used as a starting material in the ex vivo activation and expansion of NK cell products.

The objective of this study was to optimize a protocol to activate and expand NK cells by comparing different cell culture media, different aAPC and using different starting cells. The most suitable expansion protocols have been further validated within the CliniMACS Prodigy system to obtain clinical-grade NKAE cells for cancer immunotherapy. In summary, automated manufacturing clinical grade NK cells in CliniMACS Prodigy is feasible and NK cell products met the requirements established for the clinical use of this product by the Spanish Regulatory Agency.

## 2. Results

### 2.1. Optimization of Cell Growth Culture Media

#### 2.1.1. NK Cells Fold Expansion and Purity Using Different Culture Media

In order to compare fold increase and purity of NK cell expansion using different growth media, we performed a total of 21 expansion experiments with buffy coats from five different healthy donors. In these experiments, PBMCs were cocultured with previously irradiated K562-mb15-41BBL (from now on, K562mbIL15) cells in a 1:1.5 ratio in the following media: RPMI, Stem cell growth medium (SCGM), NK MACS, TexMACS GMP, all of them supplemented with 5%AB human serum and IL-2.

**Figure 1:**
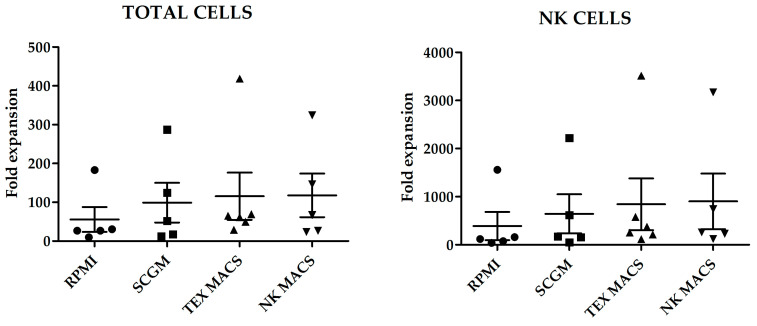
Fold increase of total cells and natural killer (NK) cells after the 21 days’ culture with the different media. Error bars show mean ± SEM. RPMI, SCGM and NK MACS *n* = 5. TexMACS *n* = 6. Geometrical symbols represent individual data of expanded cells from different donors by using RPMI (dots), SCGM (squares), TexMACS (triangles), NKMACS (inverted triangles).

Fold increase of total cells and NK cells, after 21 days culture with the different media is shown in Figure 1. Although we did not observe statistically significant differences in the number of total cells or NK cells expanded; NK MACS yielded the highest NK cells fold increase (903 ± 576.3) and RPMI medium the lowest (388 ± 292.7) (*p* = 0.45) (Figure 1). 

**Figure 2:**
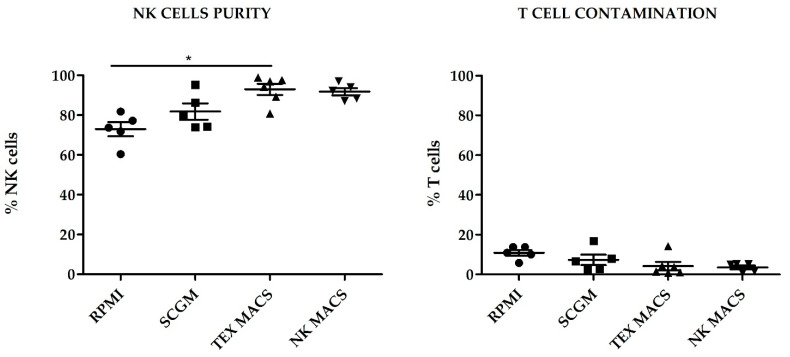
NK cell purity and T cell contamination on activated and expanded NK cells (NKAE) expanded with different culture media. Error bars show mean ± SEM. RPMI, SCGM and NK MACS *n* = 5. TexMACS *n* = 6. * *p* < 0.05. Geometrical symbols represent individual data of expanded cells from different donors by using RPMI (dots), SCGM (squares), TexMACS (triangles), NKMACS (inverted triangles).

Regarding purity, TexMACS GMP medium yielded the highest NK cell purity at day 21 (92.93% ± 2.8%), followed by NKMACS (91.75% ± 1.82%), with no significant differences between them. SCGM showed modest NK cell purity (81.79% ± 4.05%). Those cells cultured with RPMI showed the lowest NK cell purity (72.97% ± 3.6%), and the difference was statistically significant when compared to TexMACS (*p* < 0.05). NKMACS medium and TexMACS showed similar percentages of residual T cells (3.5% ± 0.96%) and (4.21% ± 2.08%), respectively, and lower than those obtained with SCGM (7.33% ± 2.62%). The highest T cell contamination was observed in those NKAE cells cultured with RPMI (10.86% ± 1.47%) although the difference with the other media was not significant (Figure 2).

Additionally, percentages of the different subpopulations, including NK bright and NK dim, and cell viability at different time points during the culture are indicated in Table S1. We did not observe differences in viability in the different NKAE cell products obtained with the different media. The percentage of bright NK cells was statistically significantly higher when RPMI was used for the expansion, compared to the other three media. Consequently, the percentage of dim NK cells was lower in those NKAE cells; however, only statistically significant differences were observed when these NKAE cells were compared to those obtained using TexMACS. No statistically significant differences were observed in the percentage of NKT or B cells expanded at day + 21.

#### 2.1.2. Expression of Activating and Inhibitory Receptors on NKAE Cells

**Figure 3:**
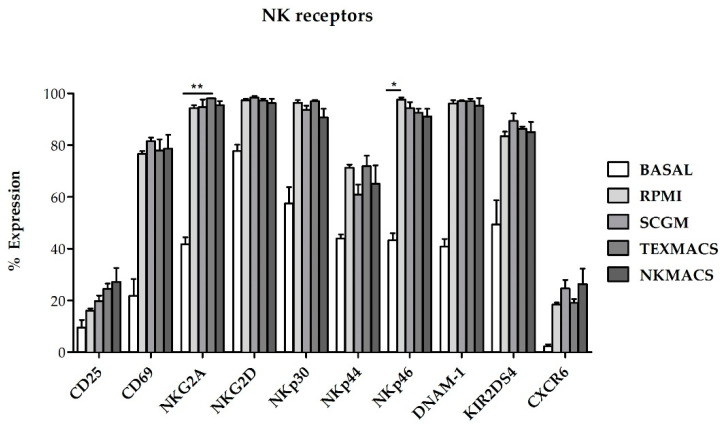
Expression of activating and inhibitory receptors on NKAE cells expanded with different culture media. The different receptors were upregulated on NKAE cells compared to basal NK cells. No differences in the expression of the analyzed receptors on NKAE cells were found when using different cell growth media. Error bars show mean ± SEM. *n* = 3 for each condition. (* *p* = 0.013, ** *p* = 0.01).

The phenotype of NKAE cells was evaluated by flow cytometry (FCM) at baseline and at day 21 following ex vivo expansion with K562mbIL15 cell line in RPMI, SCGM, NK MACS or TexMACS medium. We found that the surface expression of the functionally relevant receptors CD25, CD69, NK group-2 member D receptor (NKG2D), NKp30, NKp44, NKp46 and DNA accessory molecule 1 (DNAM-1) was upregulated in NKAE cells cultured with all media when compared with resting NK cells. However, significant differences were only observed in NKG2A and NKp46 receptors when comparisons were established between basal NK cells and NKAE cultured with TexMACS (*p* = 0.01) basal NK cells vs. NKAE RPMI, respectively (*p* = 0.013) (Figure 3).

#### 2.1.3. Cytotoxicity of NKAE Cells

**Figure 4:**
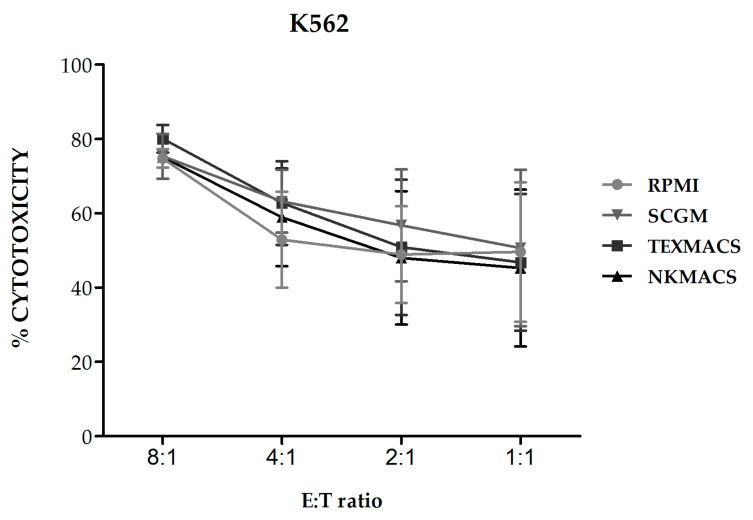
Cytotoxicity of NKAE cells expanded with different culture media against K562 cells. The NKAE cells expanded with the different culture media did not show differences in their cytolytic ability against K562 cells. Error bars show mean ± SEM. *n* = 3 for each condition.

We next evaluated the cytolytic activity of NKAE cell products obtained using the different growth media against K562 target cells and we observed no significant differences in their lytic activity (Figure 4).

### 2.2. Use of CD45RA^+^ Cells as a Source of NK Cells to Obtain NKAE

**Figure 5:**
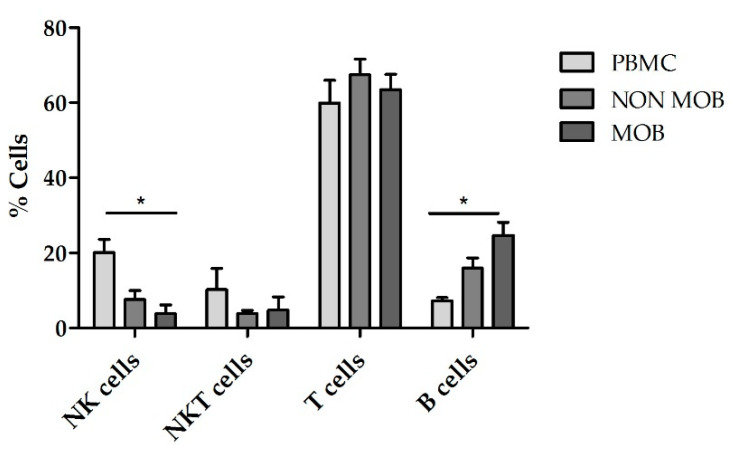
Cell subsets contained in PBMC, CD45RA^+^ cells from mobilized apheresis (mob) and CD45RA^+^ cells from non-mobilized apheresis (non-mob) before NK cell expansion. Mobilized CD45RA^+^ cells contain less NK and more B cells compared to PBMC *p* = 0.03 and *p* = 0.01 respectively. PBMC (*n* = 5), non-mob (*n* = 3) and mob (*n* = 3). Error bars show mean ± SEM. * *p* < 0.05.

**Figure 6:**
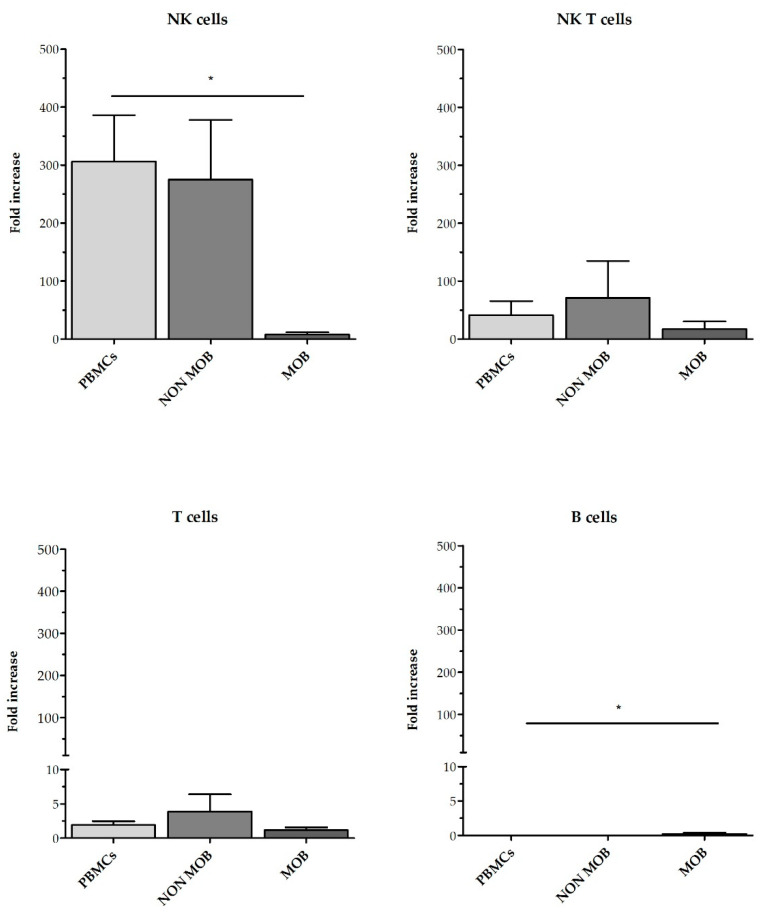
Fold increase of the different cell subsets in NKAE cells obtained from PBMC, CD45RA^+^ mobilized apheresis and CD45RA^+^ non-mobilized apheresis at day + 21. Those NKAE cells expanded from mobilized apheresis had less NK cells at the end of the expansion compared to NKAE cells from PBMC and non-mob apheresis. PBMC (*n* = 5), non-mob (*n* = 3) and mob (*n* = 3). Error bars show mean ± SEM. * *p* < 0.05.

CD45RA^+^ cells are enriched in naïve T cells and NK cells. This fraction is depleted and discarded in some HSCT procedures to remove alloreactive T cells and reduce the risk of GvHD. On the contrary, CD45RA^-^ memory T cells show decreased alloreactivity and lack naïve T cells, thus improving engraftment and protecting the recipient from infections. CD45RA^-^ cells can be infused into patients undergoing haploHSCT either as part of the graft, along with the mobilized CD34^+^ cells, or as donor lymphocyte infusions (DLI). CD45RA^+^ depleted grafts are usually obtained from healthy donors after pharmacologic mobilization of hematopoietic stem cells using G-CSF [21], although some other protocols use non-mobilized apheresis [22]. CD45RA^+^ depleted fractions for DLI are obtained after non-mobilized apheresis [23]. To evaluate the feasibility of expanding NK cells using the CD45RA^+^-wasted fraction, we performed expansions from CD45RA^+^ cells obtained after mobilized and non-mobilized apheresis and compared them with expansions using PBMCs. According to the NK cell purity data previously obtained, we used TexMACS growth medium for these expansions. The basal (preculture) composition of the different starting cell products is shown in Figure 5. CD45RA^+^ cells from mobilized apheresis showed the lowest percentage of initial NK cells purity (1.7% ± 0.6%) followed by CD45RA^+^ cells from non-mobilized apheresis (7.5% ± 2.5%) and PBMCs that had the highest basal percentage of NK cells (16.9% ± 2.7%). Total cells at the beginning of the expansion are shown in Figure S1. Fold expansion of NK cells, NKT cells, T cells and B cells after 21 days are shown in Figure 6. CD45RA^+^ mobilized fraction showed the lowest NK cells fold expansion (7.6 ± 4.2 fold); (*p* = 0.011 compared to PBMC) and the highest B cells expansion this difference was statistically significant when compared to PBMCs (306.3 ± 79.9 fold); *p* = 0.014.

### 2.3. Expansion of NKAE Cells by Using Different aAPC

**Figure 7:**
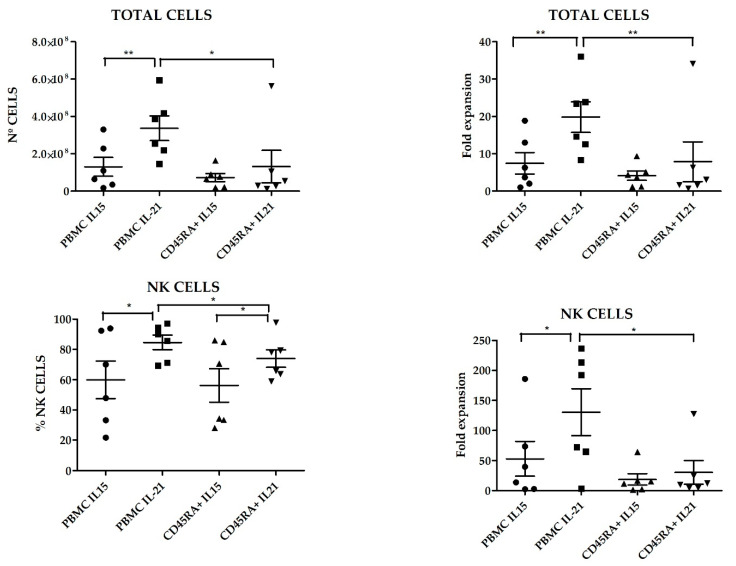
Total cell numbers, NK cell purity and fold expansion of total and NKAE cells using different NK cell sources and different artificial antigen presenting cells (aAPC) (*n* = 6 in each condition). Error bars show mean ± SEM. * *p* < 0.05, ** *p* < 0.01. Geometrical symbols represent individual data of NKAE obtained from different donors in the conditions specified in the X axis. Dots: PBMC+K562mbIL15; Squares: PBMC+ K562mbIL21, triangles: CD45RA+ K562mbIL15, inverted triangles: CD45RA+ K562mbIL21.

**Figure 8:**
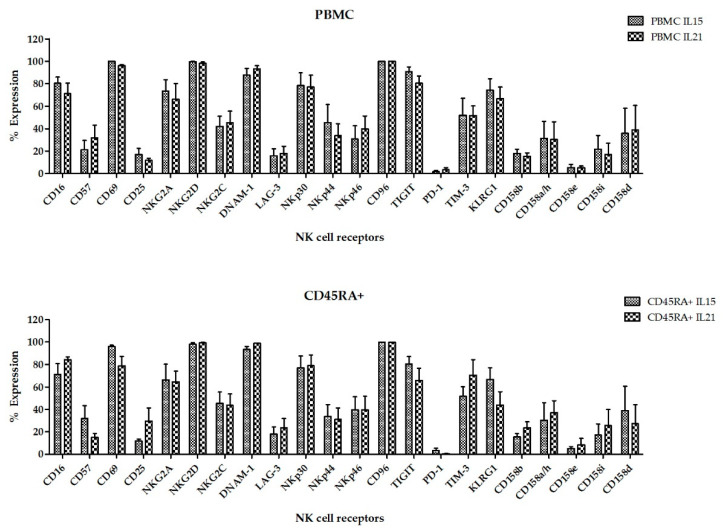
Expression of different NK cell receptors on NKAE cells expanded from PPBMC or CD45RA+ cells using K562mbIL15 or K562mbIL21 as aAPC. No differences were found in the surface expression of NK cell receptors (*n* = 4 for each condition). Error bars show mean ± SEM.

**Figure 9:**
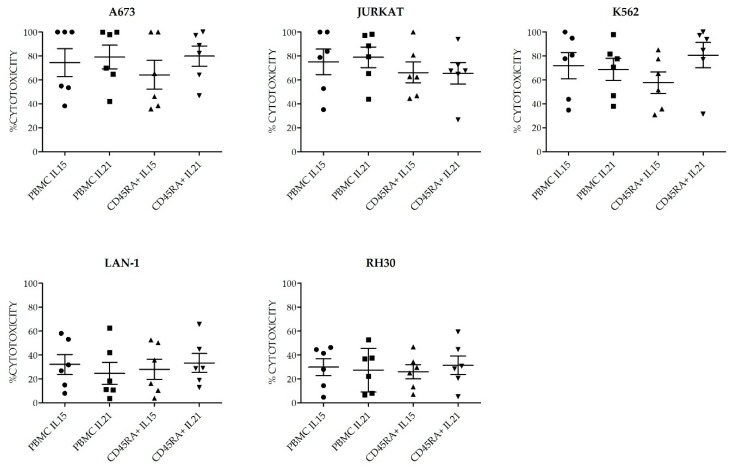
Cytotoxicity of NKAE cells expanded from PPBMC or CD45RA+ cells using K562mbIL15 or K562mbIL21 as aAPC. No differences in antitumor ability were observed among the different NKAE cell products. Error bars show mean ± SEM. E:T ratio was 2:1. Geometrical symbols represent individual data of NKAE obtained from different donors in the conditions specified in the X axis. Dots: PBMC+K562mbIL15; Squares: PBMC+ K562mbIL21, triangles: CD45RA+ K562mbIL15, inverted triangles: CD45RA+ K562mbIL21.

Once we had determined the best suited cell culture media and proven the feasibility of expanding NK cells from CD45RA^+^ fraction, we next wanted to explore if there were differences in those NKAE cells expanded with K562mbIL15 or K562-mb21-41BBL (from now on K562mbIL21) stimulatory cell lines. In these experiments, we expanded NK cells from six different donors, using PBMCs or CD45RA^+^ cells as starting material and TexMACS as expansion medium. At 21 days of culture, we evaluated total cell expansion, NK cell purity (percentage), cytotoxicity against four different pediatric tumor cell lines: A673 (sarcoma), RH30 (rhabdomyosarcoma), Jurkat (T-ALL), LAN-1 (neuroblastoma) and K562 cells as control. PBMC tended to yield higher total cell numbers and NK cell purity compared to CD45RA^+^ (2.34 × 10^8^ ± 5.04 × 10^7^ vs. 1.08 × 10^8^ ± 4.38 × 10^7^, *p* = 0.004) and (72.26% ± 25.54% vs. 65.06% ± 22.68%, *p* = 0.015) respectively. When PBMC were used as starting cells, expansion with K562mbIL21 yielded higher total cell numbers than using K562mbIL15 (3.36 × 10^8^ ± 6.63 × 10^7^ and 1.3 × 10^8^ ± 5.03 × 10^7^, respectively, *p* = 0.03) and tended to achieve higher NK cell purity (84.67 ± 4.82 vs. 59.85 ± 12.42 *p* = 0.09). When CD45RA^+^ cells were used, those expansions using K562mbIL21 tend to show higher total cells (7.31 × 10^7^ ± 2.18 × 10^7^ vs. 1.32 × 10^8^ ± 8.7 × 10^7^) and NK cell purity (73.91% ± 5.82% vs. 56.2% ± 11.11%) than those using K562mbIL15, although these differences were not statistically significant (*p* = 0.0.53 and *p* = 0.19 respectively). NKAE cells obtained after coculture with K562mbIL15 tended to yield lower cell numbers (1.02 × 10^8^ ± 2.75 × 10^7^ vs. 2.34 × 10^8^ ± 6.07 × 10^7^, *p* = 0.023) and achieved less NK cell purity (58.03% ± 7.97% vs. 79.29% ± 3.95%, *p* = 0.003) than those expanded with K562mbIL21, no matter the source of NK cells that were used (PBMC or CD45RA^+^) (Figure 7). Representative data of NK cell purity analyzed by FCM along the expansion procedure in the different conditions are shown in Figure S2. The flow cytometry analysis showed a comparable expression of surface markers in all NKAE cell products regardless of the NK cell source and the aAPC used for the expansion (Figure 8). Representative data of NK cell receptors expression in PBMC and CD45RA^+^ cells in basal conditions and in NKAE cells at day + 21 of expansion are shown in Figure S3. Consequent with the expression of activating receptors, the different NKAE cells had similar cytolytic ability against the five different tumor cell lines tested (Figure 9). 

### 2.4. Transcriptome Analysis of Basal NK Cells and Different NKAE Cell Products

To further explore the differences in gene expression of basal NK cells obtained from PBMC or CD45RA^+^ and their respective NKAE cell products expanded with different aAPC (K562mbIL15 or K562mbIL21), RNAseq analysis of two representative donors was performed.

**Figure 10:**
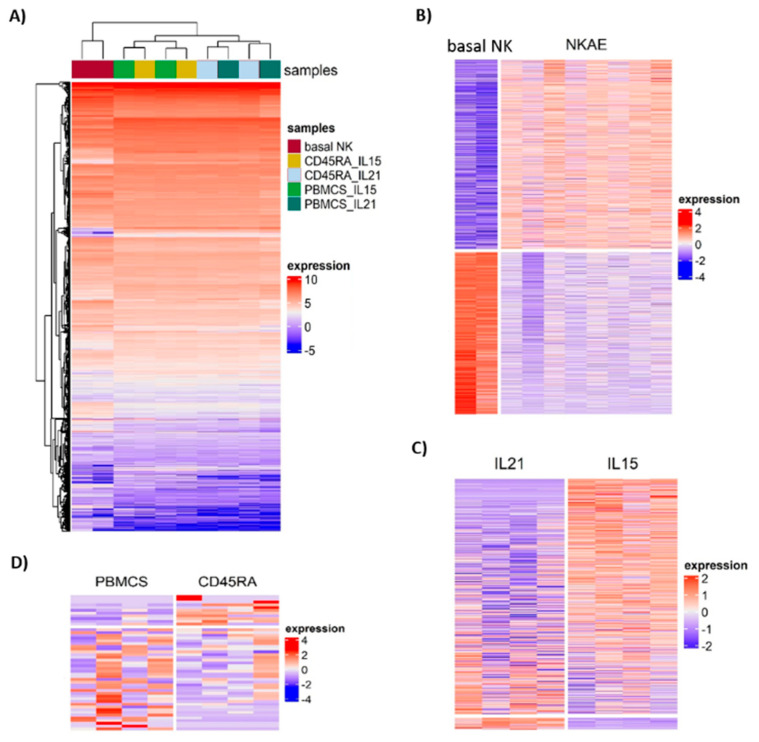
Gene expression profiles of basal NK cells and NKAE cell products. (**A**) Unsupervised hierarchical clustering of NK cells using the 15,919 genes remaining after filtering. (**B**) Heat map of normalized expression values for 2185 genes that are differentially expressed between basal NK cells and NKAE cell products. (**C**) Heat map of normalized expression values for 609 genes that are differentially expressed between IL21 and IL15-stimulated NKAE cells. (**D**) Heat map of normalized expression values for 48 genes that are differentially expressed between PBMC and CD45RA^+^-derived NKAE cells. Heat map colors correspond to gene expression as indicated in the color key: red (overexpressed) and blue (underexpressed). Each row in the heatmap represents a gene and each column, an individual sample. Deep red line: basal NK cells, green line: IL15-stimulated PBMC-derived NKAEs, yellow line: IL15-stimulated CD45RA-derived NKAEs, light blue line: IL21-stimulated CD45RA-derived NKAEs, emerald green line: IL21-stimulated PBMC-derived NKAEs.

Unsupervised hierarchical clustering of the 15,919 genes remaining after filtering divided the samples into two groups: basal NK cells and NKAE cell products (Figure 10A). NKAE cells were in turn separated into IL21 and IL15-stimulated cells, but no cluster of PBMC and CD45RA^+^-derived NKAE cells was observed. We found a total of 2185 differentially expressed genes (DEGs) in unstimulated and stimulated NK cells. Of the 2185 DEGs, 1178 were upregulated and 1007 were downregulated in stimulated NK cells at a false discovery rate (FDR) of 0.05 and log fold change (log FC) of 2 (Figure 10B). A comprehensive list of the top DEG is provided in Table S2. Kyoto Encyclopedia of Genes and Genomes (KEGG) pathway analysis showed that the DEGs were concentrated in 30 pathways, most of them related to cell growth, cell death and metabolism (cell cycle, hematopoietic cell lineage, p53 signaling pathway, pyrimidine metabolism, etc.) (Table 1). In addition, the DEG was grouped according to their associated gene ontology (GO) terms. While overexpressed genes clustered to biological processes such as cell division, DNA replication and cell proliferation, under expressed genes were involved in cell adhesion and cell migration.

Comparison of IL21 and IL15-stimulated NKAE cells led to the identification of 609 DEGs, 29 of them showing upregulation and 580 showing downregulation in IL21-stimulated NKAE cells (Figure 10C and Table S3). The number of DEGs between IL21 and IL15-stimulated NKAE cells was larger when the cell source was PBMC compared with CD45RA (547 vs. 381 DEGs). The 22 enriched pathways were related to the inflammatory response and the immune system (cytokine–cytokine receptor interaction, hematopoietic cell lineage, asthma, inflammatory bowel disease (IBD), primary immunodeficiency, IL-17 signaling pathway, etc.) (Table 1). GO term enrichment analysis showed cluster to biological processes such as immune response, inflammatory response, phagocytosis, complement activation, B cell receptor signaling pathway and cell adhesion.

Many cytokines and cytokine receptors involved in cell differentiation and activation were overexpressed under IL15 stimulation when compared with IL21 stimulation (CD5, CD4, CD8B, CD3G, CD19, CD22, CD24, CD20, CD23, CD35, IL4, IL5, CD116, CD123, CD33, CD126, CD13, IL1A, IL1B, CD121, IL9R, CD125, CD36 and CD41). Regarding the expression of NK activating and inhibitory receptors, we only identified one killer-cell immunoglobulin-like receptor (KIR) gene differentially expressed between IL21 and IL15-expanded NK cells, KIR2DL3, which was overexpressed in IL-21-expanded NK cells. Differences in the expression of leukocyte immunoglobulin like receptor B1 (LIR1), natural-killer group 2 member A (NKG2A), C (NKG2C) and D (NKG2D) and natural cytotoxicity receptors (NCRs) were not found. Concerning inhibitory checkpoint receptors, programmed cell death 1 (PD1) was overexpressed in IL15-expanded NK cells. We did not find significant changes in the expression of markers of apoptosis and proliferation such as caspase 3 (CASP3), caspase 8 (CASP8), BCL2 apoptosis regulator (BCL2), BCL2 associated X apoptosis regulator (BAX), BCL2-Like 14 apoptosis facilitator (BCL2L14), cyclin dependent kinase inhibitor 2A (CDKN2A), telomerase reverse transcriptase (TERT), MYB proto-oncogene like 2 (MYBL2), BUB1 mitotic checkpoint serine/threonine kinase (BUB1), polo like kinase 1 (PLK1), cyclin E1 (CCNE1), cyclin D1 (CCND1) and cyclin B1 (CCNB1).

Finally, a small number of DEGs was observed between PBMC and CD45RA^+^-derived NKAE cells. Of the 48 DEGs, 37 showed upregulation and 11 downregulation in PBMC-derived NKAE cells (Figure 10D and Table S4). No KEGG pathways or GO terms were significantly enriched for the DEG genes. Nevertheless, among the IL21-stimulated NKAE cells, two pathways were enriched when comparing PBMC and CD45RA-derived NKAE cells: hematopoietic cell lineage and arachidonic acid metabolism. 

### 2.5. Production of Clinical Grade NKAE Cells in CliniMACS Prodigy

After the optimization of the conditions to better expand NK cells, we wanted to test the feasibility of manufacturing these NKAE cells for clinical use. To this aim, we performed a clinical-scale completely automated expansion procedure using the CliniMACS Prodigy^®^ device (Miltenyi Biotec, Bergisch Gladbach, Germany). According to our previous results, we used non-mobilized apheresis as a starting material and TexMACS GMP-compliant medium (Miltenyi Biotec, Bergisch Gladbach, Germany). For the clinical manufacturing, GMP-compliant additional steps of CD3 depletion and CD56 selection were performed to abbreviate NK cell expansion times. Either irradiated K562mbIL15 or K562mbIL21 cells were used as aAPC. 

A total of 2–2.5 × 10^6^ of purified CD56^+^ cells were cocultured with 40 × 10^6^ of irradiated K562mbIL15 or K562mbIL21 cells, at an approximate ratio of 1:20. Viability, number of total cells, percentage and number of NK cells of starting CD56^+^ cells at days +7 and +14 of coculture are shown in Table 2. FCM data showing NK cell purity along the manufacturing process are shown in Figure S4.

On day 0, before the expansion, both CD56^+^ cells and aAPC met the acceptance criteria (sterility, mycoplasma negative and viability ≥70%).

**Figure 11:**
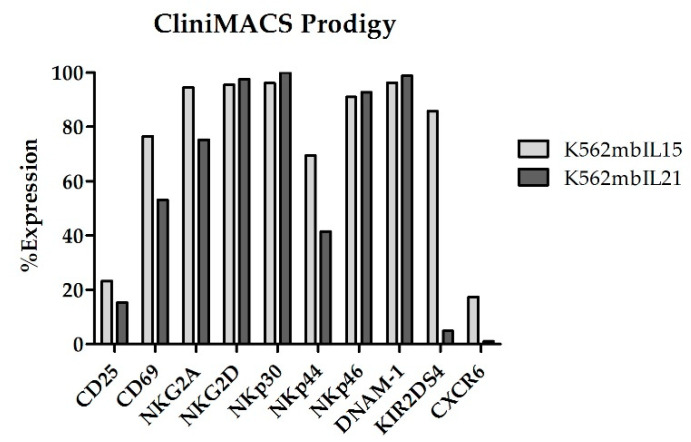
NK cell receptors are upregulated on NKAE cells manufactured in CliniMACs Prodigy.

At the end of coculture (Day +14), NKAE cells expanded in CliniMACS Prodigy showed an upregulation of all the receptors analyzed (Figure 11). 

The acceptance criteria for the manufactured NKAE cells at the end of the process included: viability ≥70%, cytotoxicity against K562 cells at an 8:1 E:T ratio ≥50%, mycoplasma negative, sterility (0 colony forming units, CFU), endotoxins ≤0.25 EU/mL, undetectable bcr/abl (absence of residual aAPC) and no overexpression of oncogenic genes C-MYC and TERT. As shown in Table 3, the manufactured NKAE cell products complied with the specifications and met the release criteria and, thus, were suitable for clinical use.

## 3. Discussion

Natural killer cells can rapidly kill tumor cells, and have been used in clinical trials to treat patients with different malignancies [24]. In this context, significant progress has been made in NK-cell based therapies, both in haploidentical stem cell transplantation (haploSCT) [25,26,27] or the non-transplant setting [28,29,30], since NK cells contribute to the graft versus leukemia/tumor (GvL/GvT) effect with no signs of GvHD [26,27,31].

The clinical use of NK cells as therapeutic weapons against cancer has some limitations: (1) these cells represent a small fraction of peripheral white blood cells and large numbers are needed to achieve clinical benefits and (2) NK cells need to be fully activated to induce tumor cell killing. Overcoming these limitations rely on the development of GMP-compliant manufacturing methods. Several protocols to obtain fully activated NK cells for clinical use have been developed. They may differ (among others) in the source of NK cells used: PBMC [32], umbilical cord blood [33] and NK-92 cell line [34]; the addition of different activating cytokines [7,35] or in the use or not of stimulating feeder cells [14,34,35,36]. 

The aims of this study were (1) to optimize in small scale NK cell activation and expansion protocol, (2) to test the feasibility of large-scaling this procedure to manufacture NKAE cells emulating a clinical application and (3) to demonstrate that the manufactured NKAE cell products met the requirements established by the Spanish Regulatory Agency for clinical use. To optimize the best cell culture growth media, we expanded NK cells by coculturing PBMC with irradiated K562mbIL15 in four different GMP-grade growth media (RPMI, SCGM, TexMACs and NK MACS). We found all the cell culture media used yielded similar numbers of total cells and NK cells. Although RPMI expanded lower numbers of cells, the differences with the rest of the media were not statistically significant. TexMACS and NK MACS showed the highest percentage of NK cells and the lowest T cell contamination, indicating they should be the preferred growth culture media to obtain the purest NKAE cell products. These results are similar to those published by Klöß et al. [19], showing NK MACs as the best culture medium when compared with X-VIVO-10, SCGM and TexMACS. However, in this publication, compared to the NK cell proliferation in the NK MACS medium, cell expansion rates in the other cell cultures containing X-VIVO-10, SCGM or TexMACS media were significantly lower. These differences could be explained by the continued use of higher concentrations of IL-2 in their experiments (1000 IU/mL vs. 10–100 IU/mL), or the use of different starting cells (purified CD56+CD3+ cells instead of total PBMC), and the coculture of starting cells with irradiated aAPC in our experiments. The lowest total cell and NK cells fold expansion that we observed in those cultures containing RPMI are in line with the observations of Duck et al. who described a better activation and expansion of NK cells when using SCGM compared to RPMI-1640 [37]. 

Once we had optimized the cell culture medium, we tested the feasibility of obtaining NKAE cells from CD45RA^+^ fraction. CD45RA^−^ cells were enriched in the central memory and effector memory T cells and had the ability to target previous pathogens or vaccines. Additionally, CD45RA^-^ cells decreased alloreactivity and lacked naïve T cells, responsible for GVH reactions. One way to protect the host from infections after HSCT is to infuse CD45RA^-^ cells as DLI [38]. CD45RA^-^ cells are obtained upon depletion of CD45RA^+^ cells. This fraction is enriched in naïve T cells and NK cells, and thus, could be a potential source of NK cells to obtain NKAE cells. When CD45RA^+^ cells from G-CSF mobilized apheresis were used to obtain NKAE cells, no expansion of either total or NK cells was observed. However, those CD45RA^+^ cells from non-mobilized apheresis yielded similar numbers of total and NK cells to those obtained from total PBMC, proving the feasibility of using this cell fraction as a source of NK cells to obtain NKAE. The inability to expand NK cells from G-CSF mobilized apheresis that we observed could be somehow expected, as the negative impact of G-CSF on NK cells mobilization and cytotoxicity has been previously reported [39,40]. In the future, if mobilization of progenitor hematopoietic cells is needed, we could consider the use of plerixafor, as it has been proved to effectively mobilize NK cells to the peripheral blood [41].

After we proved the feasibility of expanding NK cells from CD45RA^+^ fraction, we analyzed potential differences in NKAE cells obtained from total PBMC or CD45RA^+^ cells after coculture with irradiated K562mbIL15 or K562mbIL21 cells. Taking into account only the source of NK cells used, we observed a higher fold increase of total and NK cells when PBMC were used compared to CD45RA^+^ cells. Additionally, when K562mbIL21 cells were used as aAPC larger numbers of total cells and higher NK cell purity were achieved, regardless of the NK cell source used. All these data taken together suggest that coculture of PBMC with K562mbIL21 cells should be the method of choice to achieve large numbers of purified NKAE cells. The enhanced proliferation ability of K562mbIL21 expanded NK cells was demonstrated before [42]. In this report, Denman C J et al. observed that those NK cells that expanded with K562mbIL21 presented less senescence and longer telomeres than those expanded with K562mbIL15, suggesting this could be a possible mechanism to explain their sustained proliferation. Nevertheless, the NKAE cells obtained by using different NK cell sources or aAPC, showed a similar expression of NK cell receptors and comparable antitumor cytotoxicity in vitro against different tumor cell lines.

When transcriptome was analyzed, marked differences were observed in the gene expression profile of stimulated and unstimulated NK cells, with DEGs mostly related to cell growth and metabolism. These changes in gene expression reflect the activation and expansion taking place during NK stimulation. Some of these DEGs have been previously identified with high fold change when comparing expanded and unstimulated NK cells [11,43] such as ubiquitin conjugating enzyme E2 C (UBE2C), thymidine kinase 1 (TK1), aurora kinase B (AURKB), ribonucleotide reductase regulatory subunit M2 (RRM2) and ficolin 1 (FCN1), and many of them are involved in cell cycle progression. 

A high number of DEG was identified between IL21 and IL15-stimulated NKAE cells, especially when the cell source was PBMC. These results indicate that IL21 and IL15 had different effects on NK cell expansion, producing changes in the expression of different cytokines and cytokine receptors. Most of the DEG were downregulated in IL21-stimulated NKAE cells when compared with IL15-stimulated NKAE cells. Since these genes were involved in pathways such as hematopoietic cell lineage, chemokine signaling pathway, JAK-STAT signaling pathway and PI3K-Akt signaling pathway, the differential expression of these genes could promote a more undifferentiated phenotype of NK cells under IL21 stimulation, while a more activating phenotype could be associated to IL15 stimulation.

When comparing IL21 and IL15-expanded NK cells, Denman et al. [42] found similar expression profiles, with CD160 as the only differentially expressed gene. Nevertheless, only 96 genes were assessed by these authors in contrast to the 15,919 genes analyzed in this work, which explains the lack of overlap between both studies. In addition, these authors observed an increase in the proliferation of IL21-expanded NK cells, which was associated with an increased in telomere length. In the present study, although we observed a more proliferative phenotype of IL21-expanded NK cells, we did not find an increase in the expression of telomerase reverse transcriptase (TERT) and other genes involved in telomere length regulation. However, the decrease in cytokines and cytokine receptors related to cell differentiation could explain a more proliferative phenotype. The similar expression of cytotoxicity receptors observed in the transcriptome analysis between IL21 and IL15-expanded NK cells is in accordance with the comparable surface expression of NK cell receptors observed in FCM, and the functional experiments, where no significant differences in cytotoxicity were observed.

In sum, we observed a similar gene expression profile between PBMC and CD45RA^+^-derived NKAE cells, which suggests that stimulation of both cell sources give rise to NKAEs with similar phenotypes, and this hypothesis was confirmed at least in part through the analysis of the surface expression of NK cell receptors by FCM. Nevertheless, when IL21-stimulated, PBMC-derived NKAE cells showed downregulation of the hematopoietic cell lineage pathway, suggesting a more proliferative phenotype than CD45RA+-derived NKAE cells. These results were in accordance with the cell culture experiments, where PBMC-derived NKAE cells expanded more than CD45RA+-derived NKAE cells.

Once we had optimized the cell culture growth medium and the preferred NK cells source, we moved a step further to translate these protocols to the clinical and we manufactured clinical-grade NKAE cells in GMP-compliant conditions. We ran two manufacturing processes in CliniMACS Prodigy coculturing CD56^+^ cells with irradiated K562mbIL15 or its counterpart aAPC with IL21. In the clinical setting is critical to consider two important issues (1) the need to infuse the patients as early as possible and (2) to avoid undesirable GvH reactions caused by residual T cells. Thus, in an attempt to shorten the NK cell expansion times and minimize the risk of T cell contamination in the final NKAE cell products, we used already purified CD56^+^ cells instead of total PBMC as starting cells. Both protocols using either K562mbIL15 or K562mbIL21 achieved large numbers of fully activated and highly cytotoxic NKAE cells, and these manufactured NK cell products met the release criteria and complied with the specifications from the Spanish Regulatory Agency for manufacturing under aseptic conditions. However, co-culture of CD56^+^ cells with K562mbIL21 cells yielded an NK cell fold increase 2.7 times higher, and the NKAE cells showed a more potent cytolytic capacity against K562 cells than those expanded with K562mbIL15 cells. Indicating that, as we had observed in the small-scale research-grade experiments, the use of K562mbIL21 cells as aAPC could be advantageous. Nevertheless, it is important to note that only one GMP-grade large-scale manufacturing process with each cell line and using two different healthy donors was performed, for this reason, the differences that we observed could also be explained, at least in part, by the interdonor variability. In previous clinical trials carried out in our group, clinical-grade NKAE cells were obtained by manual coculture of PBMC and irradiated K562mbIL15 in IL-2+AB serum-supplemented RPMI [32]. By these means, the total cells, total NK cells and the NK cell purity (639.78 × 10^6^ ± 435.81, 515.23 × 10^6^ ± 345.03 and 79.93% ± 17.43%, respectively) were lower than those we obtained in the present study. Thus, the results reported in this manuscript could demonstrate that the use of CD56^+^ cells as the starting cells, K562mbIL21 as aAPC, TexMACS as the cell culture growth medium and the CliniMACS Prodigy device constitute a much more advantageous strategy to obtain clinical-grade NKAE cells. 

In summary, we optimized a protocol to expand large numbers of fully functional NK cells from different sources, culture media and aAPC, and we demonstrated the feasibility of clinical-scale this procedure by using the CliniMACs Prodigy device, a semiautomated closed system. The NKAE cells manufactured by these means are suitable for direct infusion to the patient or cryopreservation and could also serve as a platform for more advanced NK cell therapies such as a combination with BiKes or genetic modification to express chimeric antigen receptors (CARs).

## 4. Materials and Methods

### 4.1. Source of NK Cells and aAPC

Peripheral blood mononuclear cells (PBMCs) were isolated from buffy coats from healthy volunteers by using Ficoll–Paque gradient centrifugation. CD45RA+ fractions were obtained after magnetic enrichment using CD45RA microbeads and running “Possel” program in the AutoMACS device (both from Miltenyi Biotec) following manufacturer instructions. As the CD45RA+ fraction is a commonly discarded material from haploidentical transplantation procedures, in some cases, CD45RA+ cells were obtained from the Hematology Service at Hospital La Paz after written informed consent, in accordance with the Declaration of Helsinki and La Paz University Hospital Ethics Committee (ethical code 4917), and this study is part of an approved clinical trial with EudraCT: 2016-003578-42. Buffy coats were obtained from the Transfusions Centre of the Comunidad de Madrid upon institutional review board approval. All donors complied with the requirements regarding quality and safety for the donation, obtaining, storage, distribution and preservation of human cells and tissues under the Spanish specific regulation.

K562mbIL15 and K562mbIL21 cells were kindly provided by Prof. Campana (National University Hospital, Singapore) and Prof. Lee (Nationwide Children’s Hospital, Ohio, EEUU), respectively and irradiated with 100 Gy before coculture.

### 4.2. Expansion Procedure

For the culture medium optimization, PBMCs were cocultured with irradiated K562mbIL15 cells in a 1:1.5 ratio in the indicated growth medium: RPMI-1640 (RPMI, Lonza, Basel, Belgium), stem cell growth medium (SCGM, Cellgenix, Freiburg, Germany), NK MACS (Miltenyi Biotec, Bergisch Gladbach, Germany) or TexMACS GMP medium (TexMACS, Miltenyi Biotec, Bergisch Gladbach, Germany). All media were supplemented with 10% human AB serum (Sigma, St. Louis, MO, USA) and IL-2 (Miltenyi Biotec, Bergisch Gladbach, Germany) at 10 IU/mL for the first week and 100 IU/mL thereafter. Fresh medium was added every 2 days to a final concentration of 1–2 × 10^6^ cell/mL.

To explore the feasibility of expanding NK cells from the CD45RA+ fraction, PBMCs or CD45RA^+^ cells from mobilized or non-mobilized apheresis were cocultured with irradiated K562mbIL15 in a 1:1.5 ratio, using complete TexMACs.

To determine the best NK cell source and aAPC, either PBMC or CD45RA+ cells were cocultured with irradiated K562mbIL15 or K562mbIL21 in a 1:1.5 ratio, using complete TexMACs.

Total cell expansion, percentage of NK cells and other lymphocyte subpopulations and viability of the cultures were monitored every week by flow cytometry. Cytotoxicity of expanded NK cells was tested against different tumor target cells between days 14 and 21 of NK cell expansion.

### 4.3. Antibodies and Flow Cytometry

Lymphocyte subpopulations were determined in a Navios flow cytometer (Beckman Coulter, Brea, CA, USA) using the conjugated antibodies listed in Table S5. T lymphocytes were defined as CD45^+^CD3^+^CD56^−^, B lymphocytes as CD45^+^CD19/CD20^+^, NKT as CD45^+^CD3^+^CD56^+^ and NK cells as CD45^+^CD56^+^CD3^−^ and further subdivided in dim (CD56^dim^CD16^+^) and bright (CD56^bright^CD16^-^) subsets.

Expression of functionally relevant receptors was evaluated within the NK population. FlowJo v10.0.7 software (BD, San Jose, CA, USA) was used for data analysis.

### 4.4. In Vitro Cytotoxicity Assays

Cytotoxicity of NKAE cells was assayed between days 13 and 20 of expansion by performing conventional 4 h Europium-TDA assays. K562, Jurkat, A673, RH30 and LAN-1 cell lines were used as targets. K562, Jurkat, A673 and LAN-1 cells were purchased from American Type Culture Collection (ATCC). RH30 was kindly provided by Dr. Roma (Vall D’Hebron Institute of Research, VHIR). All cell lines were cultured following ATCC’s recommendations and routinely tested for mycoplasma. Conventional 4-h europium-TDA release assays (Perkin Elmer, Waltham, MA, USA) at different effector:target ratios (starting at 8:1) were performed as previously described [44].

The following formulas were used to calculate spontaneous and specific cytotoxicity: % specific release =  (experimental release − spontaneous release)/(maximum release  − spontaneous release)  ×  100%, spontaneous release  =  (spontaneous release-background)/(maximum release-background) × 100.

### 4.5. Gene Expression Profiling

#### 4.5.1. Library Preparation and Sequencing

NK cells from two representative donors (1 and 2) were obtained pre and post-expansion. Total RNA was isolated using the RNeasy Mini kit (QIAGEN, Hilden, Germany) according to the manufacturer’s instructions. To remove residual genomic DNA, the RNA samples were digested with DNAse I. The RNA concentration was assessed by fluorescence quantitation using Qubit 2.0 and the HS RNA assay kit (Thermo Fisher Scientific Inc., Waltham, MA, USA), the RNA purity by spectrophotometry using Nanodrop 2000 (Thermo Fisher Scientific Inc., Waltham, MA, USA) and the RNA integrity by electrophoresis using TapeStation 4200 RNA ScreenTape (Agilent Technologies, Santa Clara, CA, USA). Library preparation and RNA sequencing were performed at Nimgenetics Company (Madrid, Spain). Library samples were prepared with the TruSeq Stranded mRNA Library Prep kit (Illumina, San Diego, CA, USA) as recommended by the manufacturer. Paired-end sequencing (2 × 100 pb) was performed with NovaSeq 6000 system (Illumina, San Diego, CA, USA), with a minimum of 25 million reads per sample and read quality of 90% > Q30.

#### 4.5.2. RNA Sequencing Analysis of NK Cells

The resulting reads were aligned using HISAT2 and hg19 as a reference. Transcript assembly and quantification were achieved with StringTie and the differential gene expression analysis between the different conditions was performed with edgeR [45] using log fold change (log FC) ≥ 2 and false discovery rate (FDR) < 0.05 as the threshold. Clustered heatmaps were done with genes that have at least one count per million reads in more than one sample. The functional analysis was performed with clusterProfiler [46] using the enrichKEGG function.

### 4.6. Manufacturing of Clinical-Grade NKAE Cells

In an attempt to shorten the times to obtain clinical-grade NKAE cell products with highest NK cell purity and less T cell contamination, ready to infuse into the patients with no further processing, we purified the CD56^+^ fraction before the expansion. The ex vivo immunomagnetic purification procedure comprised CD3 depletion followed by CD56 cell selection as previously described [47]. Automated activation and expansion process were performed in CliniMACS Prodigy instrument using the CliniMACS T520 tubing set and T cell transduction (TCT) protocol. In detail, at day 0, the coculture was initiated by using 2 × 10^6^–2.5 × 10^6^ NK cells and 4 × 10^7^ K562mbIL15 or K562mbIL21 cells previously irradiated with 100 Gy. Cells were cultured in 70 mL of GMP-grade TexMACs medium supplemented with 5% human AB serum (Sigma) and 100 IU/mL of IL-2 (Miltenyi Biotec). NK cells were incubated in the culture chamber (37 °C and 5% CO_2_) in a static culture for the first week. At day +7, agitation was started and 70 mL of fresh complete medium were added to the culture. Cells were expanded for 14 days before being harvested. Sampling was performed at day +7 for process controls, including cell counts, viability, analysis of CD56^+^/CD3^−^ cell content by FCM, mycoplasma and sterility. At the end of the expansion, cells were automatically collected in 0.9% sodium chloride solution supplemented with 0.5% human serum albumin (Albutein 20%, Grifols, Barcelona, Spain), in a sterile bag. Release quality controls: total cell counts, viability, analysis of CD56+/CD3-, CD3+/CD56- and CD56+/CD3+ cell content by FCM, cytotoxicity against K562 cells, Gram staining, endotoxins, cell impurities (K562mbIL15 or K562 mbIL21) by qPCR, mycoplasma and sterility were performed at the end of the process.

#### 4.6.1. Analysis of Cell Count, Viability and NK Cell Purity

NKAE cells were counted in a CELL-DYN Emerald hematology analyzer (Abbott) and analyzed for their viability, immunophenotype and activation status by FCM as previously described.

#### 4.6.2. Cytotoxicity

Potency of manufactured NKAE cells was tested by performing Europium-TDA conventional assays against K562 cells at 8:1, 4:1, 2:1 and 1:1 E:T ratios.

#### 4.6.3. Microbiological Tests

The expansion cell products were tested for sterility, according to Eu Ph 2.6.21. The microbiological tests were developed by the Clinical Microbiology and Parasitology Service of HULP by conventional microbiology techniques. In summary, sample tests were inoculated into separate culture media and the growth of viable microorganisms was tested after several days.

#### 4.6.4. Analyses of Non-Cellular Impurities

The detection of non-cellular impurities was carried out in accordance with the methodology recommendations of Chapter 2.6.21 and 2.6.7 of the European Pharmacopeia (EuPh) for mycoplasma and Chapter 2.6.14 for endotoxins. A DNA-binding dye-based qPCR system was employed for the detection of mycoplasma DNA in cell cultures. In the final products, the levels of endotoxins were quantified by the endotoxin test Endosafe-PTS (Charles River). Both assays were developed by the Clinical Microbiology and Parasitology Service of HULP.

#### 4.6.5. Analysis of Cellular Impurities

Given that the aAPC used for NK cell activation and expansion is a tumor cell line is necessary to ensure the absence of residual K562mbIL15 or K562mbIL21 cells in the final NKAE cells products. Both aAPC contain the fusion gene BCR/ABL, so the presence of residual cells was analyzed by performing a real-time PCR (RT-PCR) for the Mbcr transcript as previously described [48].

#### 4.6.6. Genetic Tests

Genetic tests were carried out at the end of the manufacturing process.

The expression of oncogenes c-Myc and telomerase reverse transcriptase (TERT) by RT-PCR. Briefly, the total RNA was isolated using the RNeasy kit (Sigma), and reverse transcription was performed with SuperScript IV (Invitrogen, Thermofisher Scientific Inc., Waltham, MA, USA). The qRT-PCR was performed on the ABI Prism 7900HT sequence detection system (Applied Biosystems, Thermofisher Scientific Inc., Waltham, MA, USA), using the TaqMan universal PCR master mix and TaqMan gene expression assay probes (Applied Biosystems), according to the manufacturer’s specifications. The assay identification numbers were as follows: c-Myc, Hs00153408_m1; TERT, Hs00972656_m1 and GUSβ, Hs00939627_m1. The thermal cycler conditions were: 10 min at 95 °C and 40 cycles of 95 °C for 15 s followed by 60 °C for 1 min. All the reactions were performed in triplicate. The amplification data were analyzed with ABI Prism sequence detection software 2.1 (Applied Biosystems) and the relative c-MYC and TERT expression was calculated by normalization against human GUSβ expression.

To rule out chromosomal aberrations, comparative genomic hybridization (CGH) was performed in NKAE cells. DNA from the NKAE cell products was isolated using the AllPrep DNA/RNA Micro Kit (Qiagen, Hilden, Germany) and hybridized with male reference DNA (Promega Biotech, Alcobendas, Madrid, Spain) on a 60,000 oligonucleotide CGH-SNP platform (Agilent, Santa Clara, CA, USA). The data were analyzed with Agilent CGH analytics 3.4 software (Santa Clara, CA, USA), using the statistical algorithm ADAM-2 according to a sensitivity threshold of 6.0 and an average window of 0.5 Mb. Alterations in the DNA copy number were considered when at least 5 consecutive probes were altered. Probes were annotated against the human assembly GRCh37 (also known as hg19).

### 4.7. Statistical Analysis

Statistical analysis was performed using GraphPad Prism software. Results are shown as mean ± standard error of the mean (SEM). Data sets were analyzed for Gaussian distribution by using Kolmogorov–Smirnov, D-Agostino and Pearson and Shapiro–Wilk tests. Those data without normal distribution were compared with non-parametric tests. Two-tailed Student’s paired *t* test was used when cells from the same donor in different conditions were compared. For comparisons between three or more groups, a one-way ANOVA test was used to determine statistical significance. Dunn’s multiple comparisons post hoc tests were run in conjunction with one-way ANOVAs and all groups were compared with one another. When two or more variables were compared, two-way ANOVA tests followed by Bonferroni pot hoc tests to compare replicates were run. In all cases, a *p* value of <0.05 was deemed to be statistically significant.

## 5. Conclusions

In this report, we optimized a protocol to obtain NKAE cells by using four different culture growth media (RPMI, SCGM, TexMACs and NKMACs), two different NK cell sources: PBMC or CD45RA^+^ cells and two distinct irradiated aAPC (K562mbIL15 or K562mbIL21). We determined that TexMACs was the most suitable cell culture medium to expand NK cells. NK cells could be activated and expanded from those CD45RA^+^ cells obtained from non-mobilized apheresis, although the use of PBMC as the NK cell source yielded the highest numbers of purified NKAE cells. When K562mbIL21 was chosen as aAPC, the highest numbers of NKAE cells with less contamination of T cells were achieved regardless of the NK cell source used. All NKAE cells obtained from either PBMC or CD45RA+ expanded with K562mbIL15 or K562mbIL21 showed comparable antitumor ability against sarcoma, T-ALL, CML, neuroblastoma and rhabdomyosarcoma cells. Finally, we fulfilled clinical manufacturing of NKAE cells in an automated closed system CliniMACS Prodigy by using CD56^+^ cells and either irradiated K562mbIL15 or K562mbIL21. In both processes, sufficient numbers of NKAE cells with high purity and low T cell contamination were manufactured after 14 days of culture. The different release tests performed showed that manufactured NKAE cells met the requirements and specifications from the regulatory agency, and thus were suitable for clinical use.

## Figures and Tables

**Table 1:** KEGG pathways enrichment analysis by differentially expressed genes (DEGs).

	Nº of Differentially Expressed Genes	KEGG Significant Pathways
NKAE vs. basal NK cells	2185 DEG (1178 UP + 1107 DOWN)	Cell cycle, Cytokine-cytokine receptor interaction, Viral protein interaction with cytokine and cytokine receptor, Hematopoietic cell lineage, p53 signaling pathway, Cell adhesion molecules (CAMs), Inflammatory bowel disease (IBD), Pyrimidine metabolism, Transcriptional misregulation in cancer, Biosynthesis of amino acids, Glycine, serine and threonine metabolism, Prostate cancer, PI3K-Akt signaling pathway, Antifolate resistance, Asthma, Small cell lung cancer, MAPK signaling pathway, Oocyte meiosis, Human T-cell leukemia virus 1 infection, Chemokine signaling pathway, Leishmaniasis, Cellular senescence, Starch and sucrose metabolism, Tryptophan metabolism, One carbon pool by folate, Carbon metabolism, Glioma, JAK-STAT signaling pathway, Melanoma, HIF-1 signaling pathway
IL21 vs. IL15-stimulated NKAE cells	609 DEG (29 UP + 580 DOWN)	Cytokine-cytokine receptor interaction, Hematopoietic cell lineage, Asthma, Inflammatory bowel disease (IBD), Primary immunodeficiency, IL-17 signaling pathway, Viral protein interaction with cytokine and cytokine receptor, Leishmaniasis, Fc epsilon RI signaling pathway, Osteoclast differentiation, Transcriptional misregulation in cancer, Rheumatoid arthritis, Protein digestion and absorption, Chemokine signaling pathway, T cell receptor signaling pathway, Th17 cell differentiation, JAK-STAT signaling pathway, Arachidonic acid metabolism, Malaria, NF-kappa B signaling pathway, Cell adhesion molecules (CAMs), Ether lipid metabolism
PBMC vs. CD45RA+ derived NKAE cells	48 DEG (37 UP + 11 DOWN)	-

**Table 2:** Characteristics of CD56 starting cells and expanded NKAE cells a day +7 and +14 (end of culture). (*) Viability measured in CD45+ cells. (**) At day 0, analysis was performed in CD56^+^ cells before coculture with K562mbIL15 cells.

**K562mbIL15**	% Viability (*)	**% NK Cells**	**% T Cells**	**Total Cells**	**Total NK Cells**
Day 0 (**)	96	87	22.8	2.9 × 10^6^	2.5 × 10^6^
Day +7	95	87.1	1	80 × 10^6^	76 × 10^6^
Day +14	97	97.1	1.2	654 × 10^6^	635 × 10^6^
**K562mbIL21**	% Viability (*)	**% NK Cells**	**% T Cells**	**Total Cells**	**Total NK Cells**
Day 0 (**)	96	77	14.8	3.4 × 10^6^	2 × 10^6^
Day +7	99.3	90.7	3.5	107.5 × 10^6^	97.5 × 10^6^
Day +14	97	93	1.6	1498 × 10^6^	1393 × 10^6^

**Table 3:** The manufactured NKAE cells met the established acceptance criteria.

aAPC	Sterility	Mycoplasma	Endotoxins	c-myc/tert	Bcr/abl	Cytotoxicity
K562mbIL15	0 CFU	Negative	<0.01 EU/mL	No expression	0%	79%
K562mbIL21	0 CFU	Negative	<0.03 EU/mL	No expression	0%	100%

## Data Availability

The RNAseq data discussed in this publication have been deposited in NCBI’s Gene Expression Omnibus (Edgar et al., 2002, doi:10.1093/nar/30.1.207) and are accessible through GEO Series accession number GSE165849 (https://www.ncbi.nlm.nih.gov/geo/query/acc.cgi?acc=GSE165849).

## Supplementary Materials

The following are available online at https://www.mdpi.com/2072-6694/13/3/577/s1, Figure S1: Total cell numbers contained in PBMC, CD45RA+ cells from mobilized and no-mobilized apheresis. Figure S2: Representative FCM data of NK cell purity observed along the expansion in the different conditions. Figure S3: Representative data of the expression of NK cell receptors expressed in PBMC, CD45RA+ cells and in NKAEs at day +21. Figure S4: NK cell purity along the manufacturing protocol in CliniMACS Prodigy. Table S1: Total cell number, viability and percentages of lymphocyte subsets found in NKAEs at different time points cultured in RPMI, SCGM, TexMACS or NK MACS. Table S2: Top 100 overexpressed and 100 underexpressed genes in stimulated NK cells when compared with unstimulated NK cells, ranked by fold change. Table S3: Top 25 overexpressed and 25 underexpressed genes in IL21-stimulated NK cells when compared with IL15-stimulated NK cells, ranked by fold change, Table S4: DEG between PBMC- and CD45RA+-derived NK cells ranked by fold change.
