# Supplementary material for: Optimizing the Procedure to Manufacture Clinical-Grade NK Cells for Adoptive Immunotherapy
Source: Cancers (Basel). 2021 Feb 2;13(3):577. doi: 10.3390/cancers13030577 (PMC7867223; doi:10.3390/cancers13030577)
Supplement: Supplementary file 1 [file cancers-13-00577-s001.zip › ST1 modified.docx]

| **RPMI** | **DAY 0** | **DAY 7** | **DAY 14** | **DAY 21** |
| --- | --- | --- | --- | --- |
| **Total cells (x10^6^)** | 3.02 | 8.64 | 89.06 | 123.84 |
| **NK (%)** | 17.46 | 52.92 | 65.43 | 72.97 |
| **NK (x10^6^)** | 0.55 | 4.67 | 66.55 | 94.17 |
| **NK dim (%)** | 87.08 | 92.39 | 89.63 | 89.49 |
| **NKdim(x10^6^)(*)** | 0.49 | 4.54 | 62.37 | 86.94 |
| **NK bright (%)(*)** | 8.68 | 8.06 | 7.71 | 9.11 |
| **NK bright(x10^6^)** | 0.04 | 0.12 | 3.55 | 6.08 |
| **LT (%)** | 64.00 | 31.83 | 15.67 | 10.86 |
| **LT (x10^6^)** | 1.91 | 1.02 | 10.33 | 14.25 |
| **NKT (%)** | 9.10 | 5.91 | 9.78 | 10.72 |
| **NKT (x10^6^)** | 0.30 | 0.17 | 5.83 | 9.87 |
| **LB (%)** | 7.78 | 2.80 | 0.57 | 0.36 |
| **LB (x10^6^)** | 0.22 | 0.07 | 0.19 | 0.12 |
| **Viability (%)** | 99.08 | 92.75 | 93.89 | 92.40 |
| **SCGM** | **DAY 0** | **DAY 7** | **DAY 14** | **DAY 21** |
| **Total cells (x10^6^)** | 5.02 | 17.79 | 191.24 | 266.03 |
| **NK (%)** | 16.04 | 50.13 | 80.56 | 81.79 |
| **NK (x10^6^)** | 0.79 | 8.77 | 158.38 | 220.00 |
| **NK dim (%)** | 89.95 | 94.27 | 97.39 | 95.52 |
| **NKdim(x10^6^)** | 19.25 | 6.68 | 153.74 | 211.77 |
| **NK bright (%)(*)** | 9.89 | 5.79 | 2.37 | 3.88 |
| **NK bright(x10^6^)** | 0.08 | 0.45 | 4.15 | 6.89 |
| **LT (%)** | 65.94 | 28.58 | 10.21 | 8.14 |
| **LT (x10^6^)** | 3.45 | 4.73 | 16.44 | 24.32 |
| **NKT (%)** | 2.68 | 5.31 | 5.43 | 8.17 |
| **NKT (x10^6^)** | 0.14 | 0.86 | 7.48 | 17.36 |
| **LB (%)** | 8.10 | 1.43 | 0.04 | 0.00 |
| **LB (x10^6^)** | 0.33 | 0.24 | 0.04 | 0.00 |
| **Viability (%)** | 98.84 | 92.76 | 89.58 | 85.70 |
| **TexMACS** | **DAY 0** | **DAY 7** | **DAY 14** | **DAY 21** |
| **Total cells (x10^6^)** | 3.07 | 53.60 | 182.09 | 269.26 |
| **NK (%)** | 18.33 | 74.31 | 88.94 | 92.93 |
| **NK (x10^6^)** | 0.59 | 51.42 | 152.91 | 240.94 |
| **NK dim (%)(*)** | 88.32 | 97.47 | 96.74 | 97.82 |
| **NKdim(x10^6^)** | 0.52 | 50.16 | 147.18 | 234.30 |
| **NK bright (%)(*)** | 8.32 | 2.56 | 3.08 | 2.29 |
| **NK bright(x10^6^)** | 0.04 | 1.29 | 5.15 | 5.85 |
| **LT (%)** | 61.79 | 14.66 | 5.94 | 4.21 |
| **LT (x10^6^)** | 1.87 | 4.23 | 19.33 | 19.55 |
| **NKT (%)** | 8.70 | 2.56 | 2.57 | 2.22 |
| **NKT (x10^6^)** | 0.29 | 0.63 | 4.54 | 5.41 |
| **LB (%)** | 8.24 | 0.35 | 0.00 | 0.00 |
| **LB (x10^6^)** | 0.24 | 0.06 | 0.00 | 0.00 |
| **Viability (%)** | 99.16 | 92.21 | 93.48 | 91.43 |
| **NKMACS** | **DAY 0** | **DAY 7** | **DAY 14** | **DAY 21** |
| **Total cells (x10^6^)** | 5.02 | 24.00 | 273.98 | 336.81 |
| **NK (%)** | 16.04 | 67.17 | 87.50 | 91.75 |
| **NK (x10^6^)** | 0.79 | 11.91 | 238.61 | 313.47 |
| **NK dim (%)** | 89.95 | 92.57 | 96.93 | 96.39 |
| **NKdim(x10^6^)** | 19.25 | 28.89 | 216.71 | 278.78 |
| **NK bright (%)(*)** | 9.89 | 6.90 | 2.58 | 2.77 |
| **NK bright(x10^6^)** | 0.08 | 0.70 | 7.07 | 10.10 |
| **LT (%)** | 65.94 | 16.25 | 4.24 | 3.50 |
| **LT (x10^6^)** | 3.45 | 2.63 | 11.57 | 11.64 |
| **NKT (%)** | 2.68 | 3.97 | 5.27 | 2.45 |
| **NKT (x10^6^)** | 0.14 | 0.56 | 16.69 | 6.18 |
| **LB (%)** | 8.10 | 1.02 | 0.02 | 0.00 |
| **LB (x10^6^)** | 0.33 | 0.17 | 0.03 | 0.00 |
| **Viability (%)** | 98.84 | 90.82 | 88.12 | 87.24 |

**Supplementary table 1.** Total cell number, viability and percentages of lymphocyte subsets found in NKAEs at different time points cultured in RPMI, SCGM, TexMACS or NK MACS.
