# Supplementary material for: Optimizing the Procedure to Manufacture Clinical-Grade NK Cells for Adoptive Immunotherapy
Source: Cancers (Basel). 2021 Feb 2;13(3):577. doi: 10.3390/cancers13030577 (PMC7867223; doi:10.3390/cancers13030577)
Supplement: Supplementary file 1 [file cancers-13-00577-s001.zip › ST3.docx]

**Supplementary Table 3.** Top 25 overexpressed and 25 underexpressed genes in IL21-stimulated NK cells when compared with IL15-stimulated NK cells, ranked by fold change.

| **Overexpressed genes in IL21-stimulated NK cells** | | | **Underexpressed genes in IL21-stimulated NK cells** | | |
| --- | --- | --- | --- | --- | --- |
| **Genes** | **logFC** | **FDR** | **Genes** | **logFC** | **FDR** |
| BIVM-ERCC5 | 9,4 | 1,20E-12 | CD24 | 10,4 | 2,55E-27 |
| SDK2 | 9,3 | 2,31E-15 | CPA3 | 10,3 | 1,69E-29 |
| KIF19 | 7,7 | 4,33E-13 | IGHE | 10,2 | 4,03E-24 |
| C8orf44-SGK3 | 7,6 | 0,00200444 | IGFBP5 | 10,2 | 6,78E-24 |
| KIAA0408 | 7,6 | 0,00257796 | SRPX | 10,1 | 8,64E-23 |
| IGFBP2 | 6,5 | 4,09E-20 | IGHE_1 | 10,0 | 4,37E-21 |
| AS3MT | 6,3 | 4,41E-08 | TRAV26-2 | 9,7 | 2,70E-16 |
| CXXC4 | 5,5 | 0,0027352 | PLA2G4A | 9,5 | 1,08E-13 |
| PAK6 | 5,4 | 8,29E-14 | KCNK13 | 9,4 | 3,89E-13 |
| PPFIA4 | 5,0 | 3,27E-21 | BMP2 | 9,1 | 5,63E-16 |
| RBPMS2 | 5,0 | 0,04616822 | LHFPL2 | 8,9 | 2,69E-14 |
| PODN | 4,8 | 0,00195849 | TFPI2 | 8,8 | 2,19E-08 |
| CSF1R | 3,9 | 0,00100804 | IGHG4 | 8,6 | 1,53E-06 |
| TM4SF19-TCTEX1D2 | 3,8 | 0,04579934 | TRBV7-8_1 | 8,5 | 1,70E-31 |
| FLJ31356 | 3,7 | 0,04692234 | IGHG4_1 | 8,5 | 3,91E-06 |
| AK4 | 3,3 | 1,00E-12 | ANGPT1 | 8,5 | 1,74E-06 |
| C15orf48 | 3,1 | 8,41E-09 | RRAGD | 8,5 | 1,50E-08 |
| PFKFB4 | 2,8 | 4,08E-09 | AICDA | 8,3 | 2,02E-05 |
| LINC01504 | 2,8 | 0,02695302 | IL22 | 8,2 | 4,46E-05 |
| MIR210HG | 2,6 | 6,20E-08 | IGHG3 | 8,2 | 1,04E-14 |
| PTK7 | 2,5 | 0,00022898 | IGKV1D-39 | 8,2 | 5,64E-06 |
| SLCO2B1 | 2,5 | 0,00068864 | TRBV7-8 | 8,2 | 4,63E-32 |
| GNAL | 2,3 | 2,89E-05 | TCN1 | 8,2 | 8,62E-05 |
| ATP6V1G2-DDX39B_2 | 2,3 | 0,01942776 | TRBV11-3 | 8,2 | 0,00010317 |
| CERCAM | 2,3 | 1,50E-11 | F13A1 | 8,1 | 1,47E-18 |
