# Supplementary material for: Optimizing the Procedure to Manufacture Clinical-Grade NK Cells for Adoptive Immunotherapy
Source: Cancers (Basel). 2021 Feb 2;13(3):577. doi: 10.3390/cancers13030577 (PMC7867223; doi:10.3390/cancers13030577)
Supplement: Supplementary file 1 [file cancers-13-00577-s001.zip › ST4.docx]

**Supplementary Table 4.** DEG between PBMC- and CD45RA+-derived NK cells ranked by fold change.

| **Overexpressed genes in PBMC-derived NK cells** | | | **Underexpressed genes in PBMC-derived NK cells** | | |
| --- | --- | --- | --- | --- | --- |
| **Genes** | **logFC** | **FDR** | **Genes** | **logFC** | **FDR** |
| HBD | 10,6 | 4,82E-30 | C8orf44-SGK3 | 7,6 | 0,01735948 |
| BIVM-ERCC5 | 9,4 | 3,42E-11 | KIAA0408 | 7,6 | 0,0210561 |
| TRBV11-3 | 8,2 | 0,00107294 | LIMS3 | 7,5 | 0,04101822 |
| STC2 | 8,0 | 0,00228884 | BLOC1S5-TXNDC5 | 5,8 | 3,04E-08 |
| IGFBP2 | 7,8 | 1,53E-21 | ARHGAP8 | 5,6 | 0,01019331 |
| MS4A3 | 7,8 | 0,00304871 | NPHP3-ACAD11 | 3,4 | 6,74E-09 |
| HBG1 | 6,5 | 4,96E-05 | TRBC2 | 3,4 | 2,45E-12 |
| MPO | 6,4 | 3,11E-05 | PAK6 | 2,9 | 2,83E-05 |
| MIR34AHG | 5,3 | 1,38E-20 | PHOSPHO2-KLHL23 | 2,8 | 1,37E-06 |
| SDK2 | 5,2 | 2,03E-08 | FHAD1-AS1 | 2,5 | 0,04399563 |
| IL22 | 5,1 | 0,03577312 | KIF19 | 2,2 | 0,02463904 |
| HBG2 | 4,7 | 6,25E-09 |  |  |  |
| CD36 | 4,3 | 0,017237 |  |  |  |
| FCER1A | 4,3 | 3,07E-07 |  |  |  |
| AK4 | 4,2 | 2,87E-17 |  |  |  |
| CPA3 | 3,9 | 4,74E-11 |  |  |  |
| HDC | 3,7 | 8,32E-12 |  |  |  |
| TRGV8 | 3,6 | 2,00E-09 |  |  |  |
| PPFIA4 | 3,5 | 3,73E-11 |  |  |  |
| TPSAB1 | 3,5 | 4,49E-09 |  |  |  |
| TPSB2 | 3,3 | 9,98E-07 |  |  |  |
| CR1 | 3,3 | 0,00227761 |  |  |  |
| GCSAML | 3,2 | 0,04248901 |  |  |  |
| GATA2 | 3,2 | 3,04E-08 |  |  |  |
| NFE2 | 3,0 | 0,00088198 |  |  |  |
| IGLC2 | 2,9 | 8,64E-08 |  |  |  |
| DHRS2 | 2,8 | 0,02727151 |  |  |  |
| SPOCK1 | 2,8 | 0,00183292 |  |  |  |
| KLHL23 | 2,7 | 1,37E-06 |  |  |  |
| PFKFB4 | 2,6 | 4,47E-07 |  |  |  |
| MIR210HG | 2,6 | 1,86E-06 |  |  |  |
| ITGA2B | 2,5 | 3,11E-05 |  |  |  |
| IGLC3 | 2,5 | 9,32E-06 |  |  |  |
| SLC45A3 | 2,2 | 0,00057454 |  |  |  |
| SLC40A1 | 2,1 | 0,0027525 |  |  |  |
| C15orf48 | 2,1 | 0,00107294 |  |  |  |
| IGHA1_1 | 2,0 | 9,25E-06 |  |  |  |
