# Supplementary material for: Optimizing the Procedure to Manufacture Clinical-Grade NK Cells for Adoptive Immunotherapy
Source: Cancers (Basel). 2021 Feb 2;13(3):577. doi: 10.3390/cancers13030577 (PMC7867223; doi:10.3390/cancers13030577)
Supplement: Supplementary file 1 [file cancers-13-00577-s001.zip › ST5.docx]

| **Specificity** | **Clone** | **Isotype** | **Fluorochrome** | **Manufacturer** |
| --- | --- | --- | --- | --- |
| CD3 | HIT3a | Mouse IgG2a | PE-Cy7 | Biolegend |
| CD3 | REA613 | Rec. Human IgG1 | Viogreen | Miltenyi Biotec |
| CD16 | 3G8 | Mouse IgG1 | APC-Cy7 | BD Pharmingen |
| CD19 | J3-J119 | Mouse IgG1 | PE | Beckman Coulter |
| CD20 | B9E9 | Mouse IgG2a | PE | Beckman Coulter |
| CD25 | M-A251 | Mouse IgG1 | FITC | BD Pharmingen |
| CD45 | J33 | Mouse IgG1 | FITC | Beckman Coulter |
| CD45 | HI30 | Mouse IgG1 | BV510 | BD Horizon |
| CD45RA | HI100 | Mouse IgG2b | FITC | BD Pharmingen |
| CD56 | B159 | Mouse IgG1 | APC | BD Pharmingen |
| CD56 | B159 | Mouse IgG1 | Alexa Fluor 700 | BD Pharmingen |
| CD56 | NCAM16.2 | Mouse IgG2b | BV421 | BD Horizon |
| CD57 | TB03 | Mouse IgM | FITC | Miltenyi Biotec |
| CD69 | FN50 | Mouse IgG1 | PE | Miltenyi Biotec |
| CD69 | FN50 | Mouse IgG1 | BV421 | Biolegend |
| CD96 | REA195 | Rec. Human IgG1 | APC | Miltenyi Biotec |
| CD158a/h | EB6B | Mouse IgG1 | APC | Beckman Coulter |
| CD158b | CH-L | Mouse IgG2b | FITC | BD Pharmingen |
| CD158d | REA768 | Rec. Human IgG1 | APC | Miltenyi Biotec |
| CD158e | DX9 | Mouse IgG1 | FITC | Miltenyi Biotec |
| CD158i | JJC11.6 | Mouse IgG1 | PE | Miltenyi Biotec |
| NKG2A | 131411 | Mouse IgG2a | PE | R&D Systems |
| NKG2A | REA110 | Rec. Human IgG1 | Viobright FITC | Miltenyi Biotec |
| NKG2C | REA205 | Rec. Human IgG1 | PE | Miltenyi Biotec |
| NKG2D | BAT221 | Mouse IgG1 | PE | Miltenyi Biotec |
| CXCR6 | 56811 | Mouse IgG2b | PE | R&D Systems |
| NKp30 | AF29-4D12 | Mouse IgG1 | PE | Miltenyi Biotec |
| NKp44 | 2.29 | Mouse IgG1 | PE | Miltenyi Biotec |
| NKp46 | 9E2 | Mouse IgG1 | FITC | BIO RAD |
| DNAM-1 | 102511 | Mouse IgG1 | PE | R&D Systems |
| DNAM-1 | DX11 | Mouse IgG1 | PE | BD Pharmingen |
| PD-1 | EH12.1 | Mouse IgG1 | PE-CF594 | BD Horizon |
| TIM-3 | REA635 | Rec. Human IgG1 | PE-Vio770 | Miltenyi Biotec |
| KLRG1 | SA231A2 | Mouse IgG2a | BV421 | Biolegend |
| LAG-3 | REA351 | Rec. Human IgG1 | PE | Miltenyi Biotec |
| TIGIT | REA1004 | Rec. Human IgG1 | PE | Miltenyi Biotec |
| 7-AAD |  |  |  | BD Pharmingen |

**Supplementary Table 5.** List of labeled antibodies used in this study.
