# Supplementary figures and images for: Optimizing the Procedure to Manufacture Clinical-Grade NK Cells for Adoptive Immunotherapy
Source: Cancers (Basel). 2021 Feb 2;13(3):577. doi: 10.3390/cancers13030577 (PMC7867223; doi:10.3390/cancers13030577)

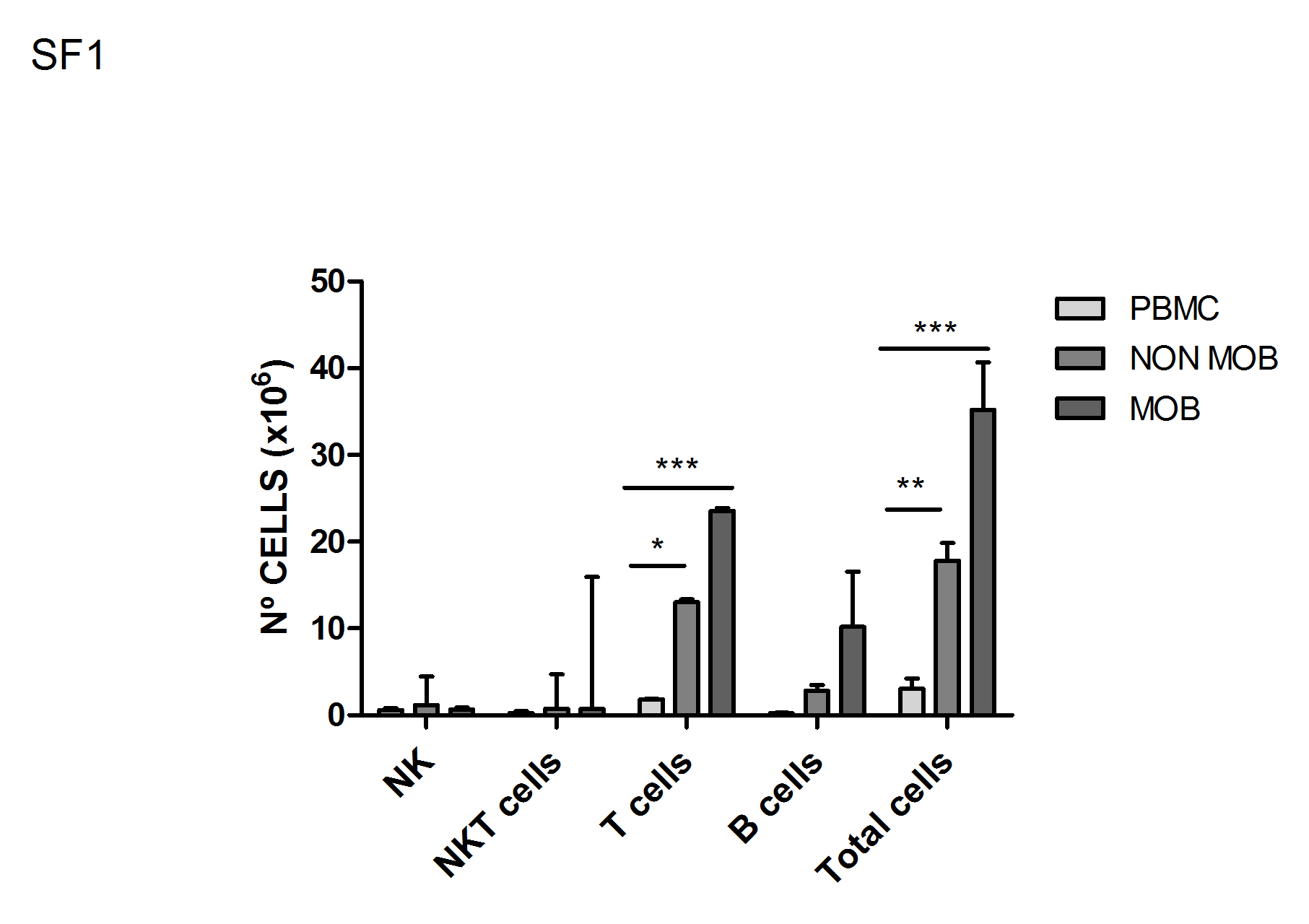

Supplement: Supplementary file 1 [file cancers-13-00577-s001.zip › SF1.jpg]

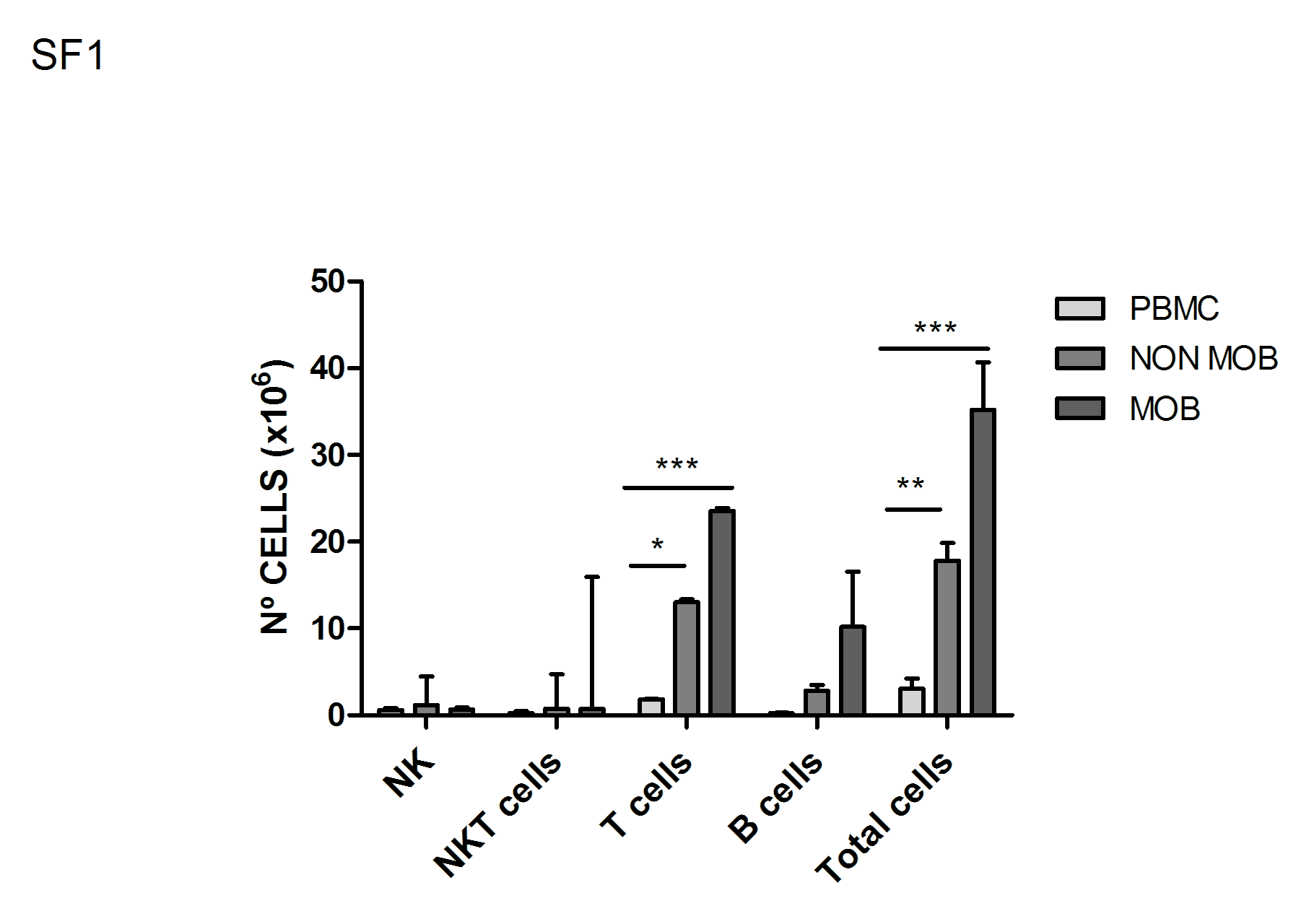

Supplement: Supplementary file 1 [file cancers-13-00577-s001.zip › SF1.tif]

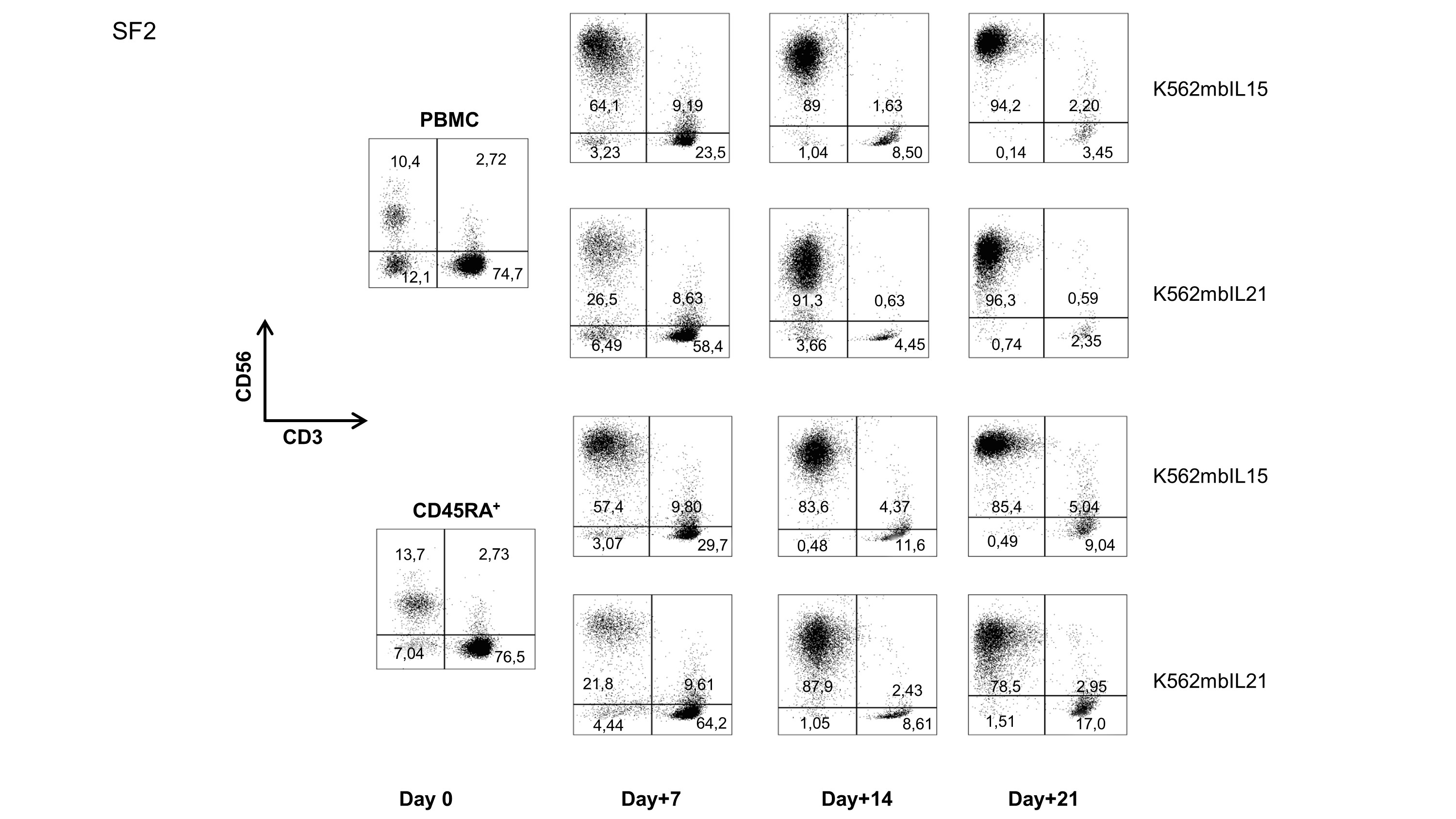

Supplement: Supplementary file 1 [file cancers-13-00577-s001.zip › SF2.jpg]

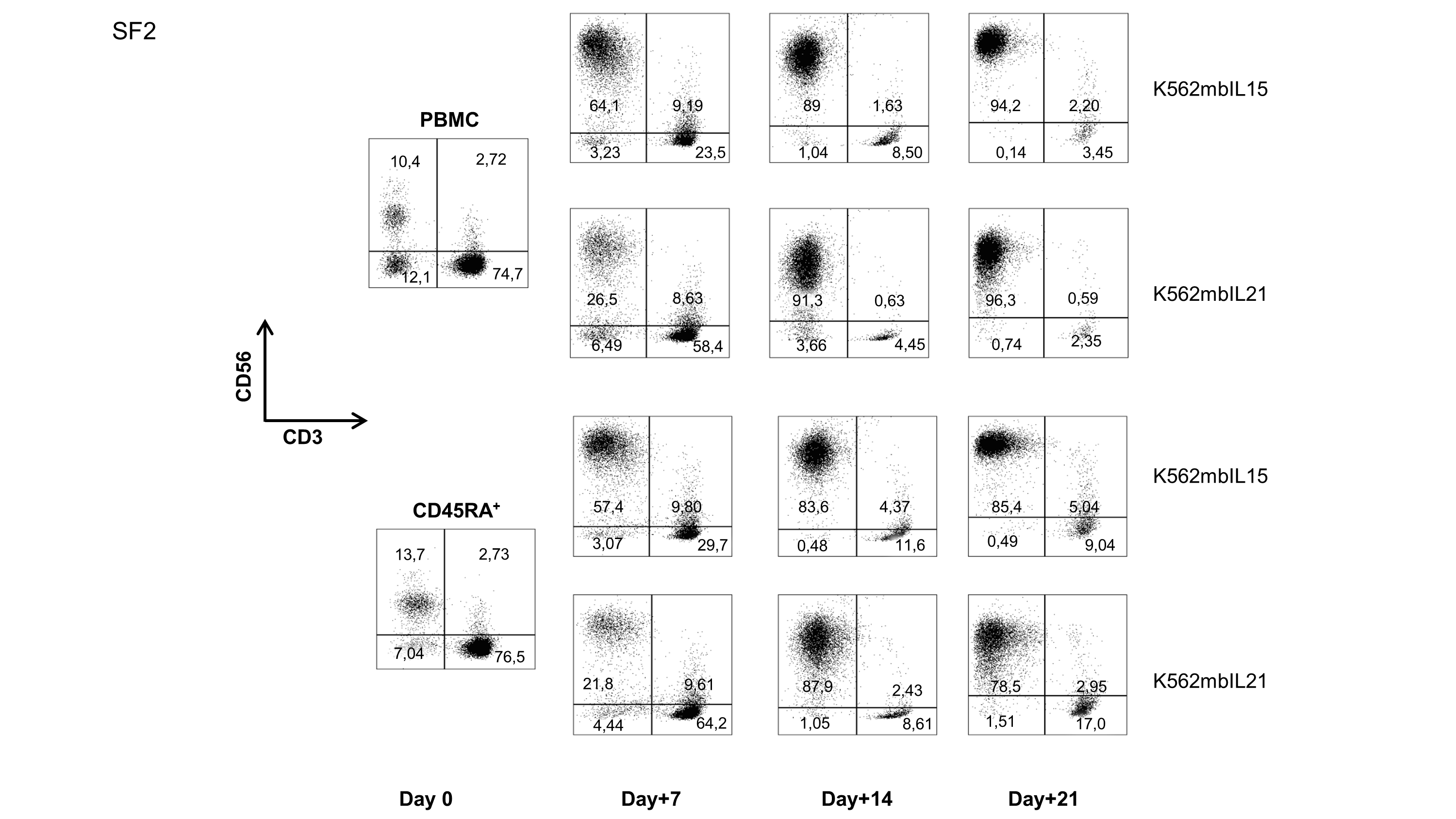

Supplement: Supplementary file 1 [file cancers-13-00577-s001.zip › SF2.tif]

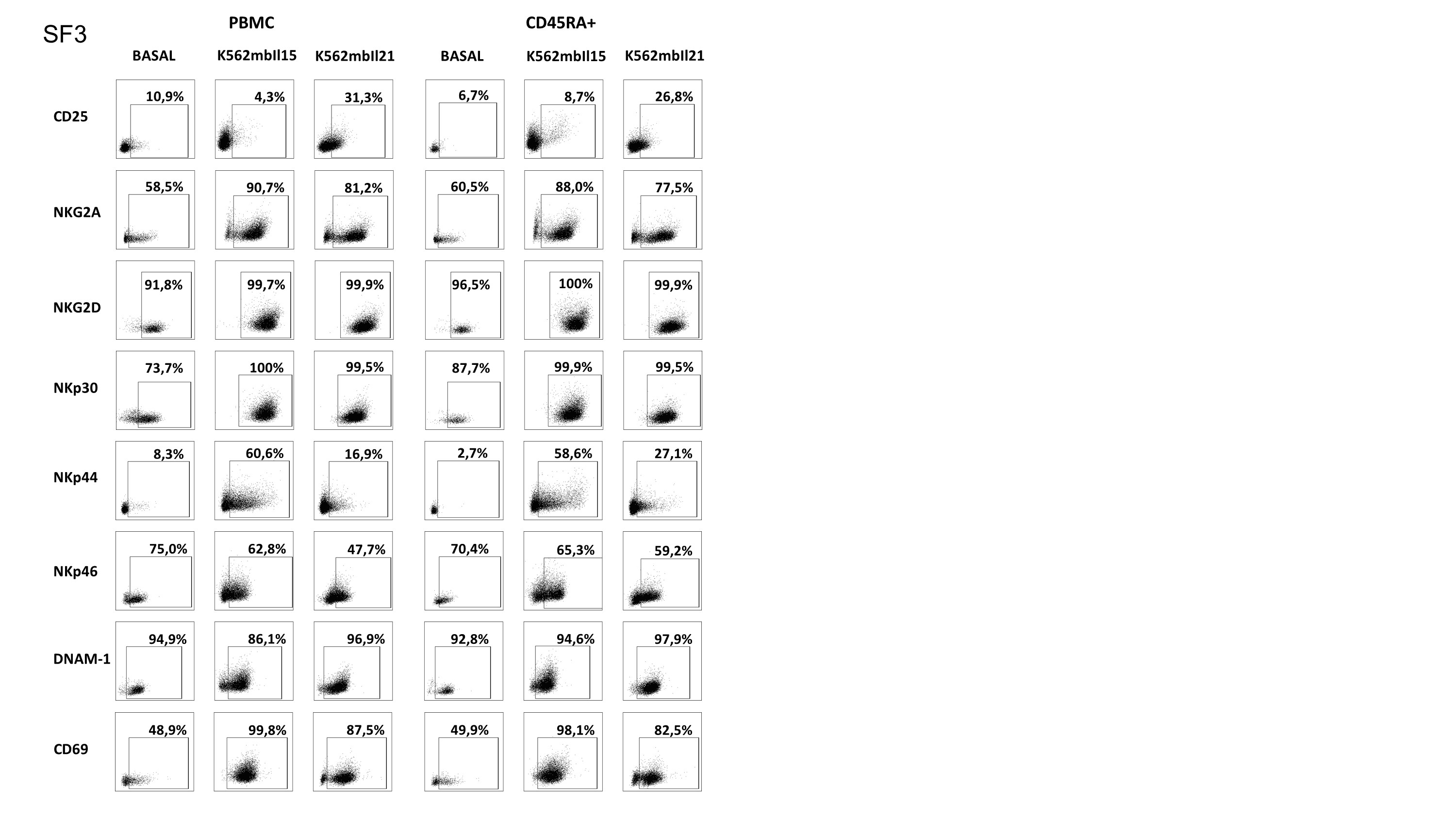

Supplement: Supplementary file 1 [file cancers-13-00577-s001.zip › SF3.jpg]

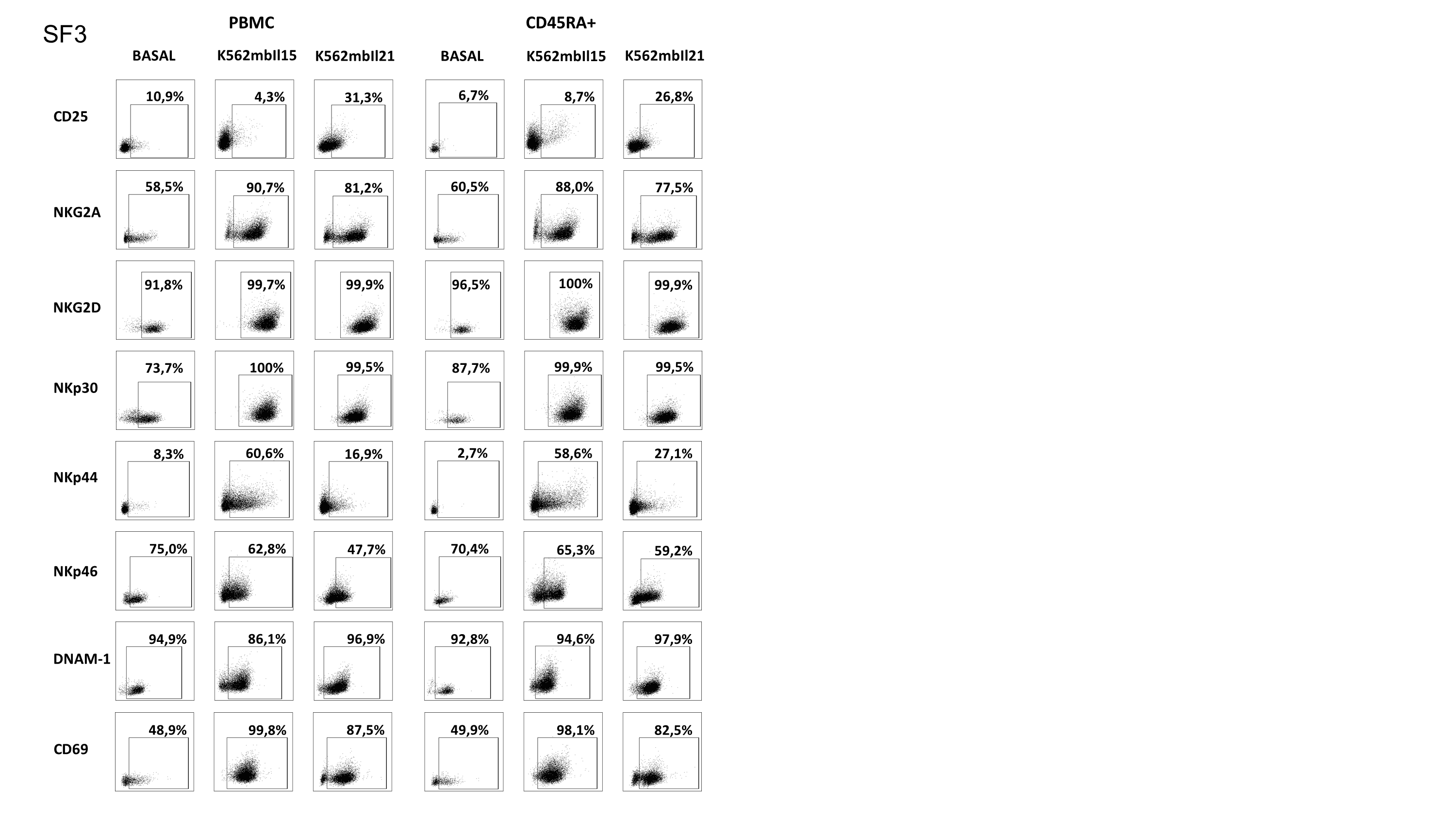

Supplement: Supplementary file 1 [file cancers-13-00577-s001.zip › SF3.tif]

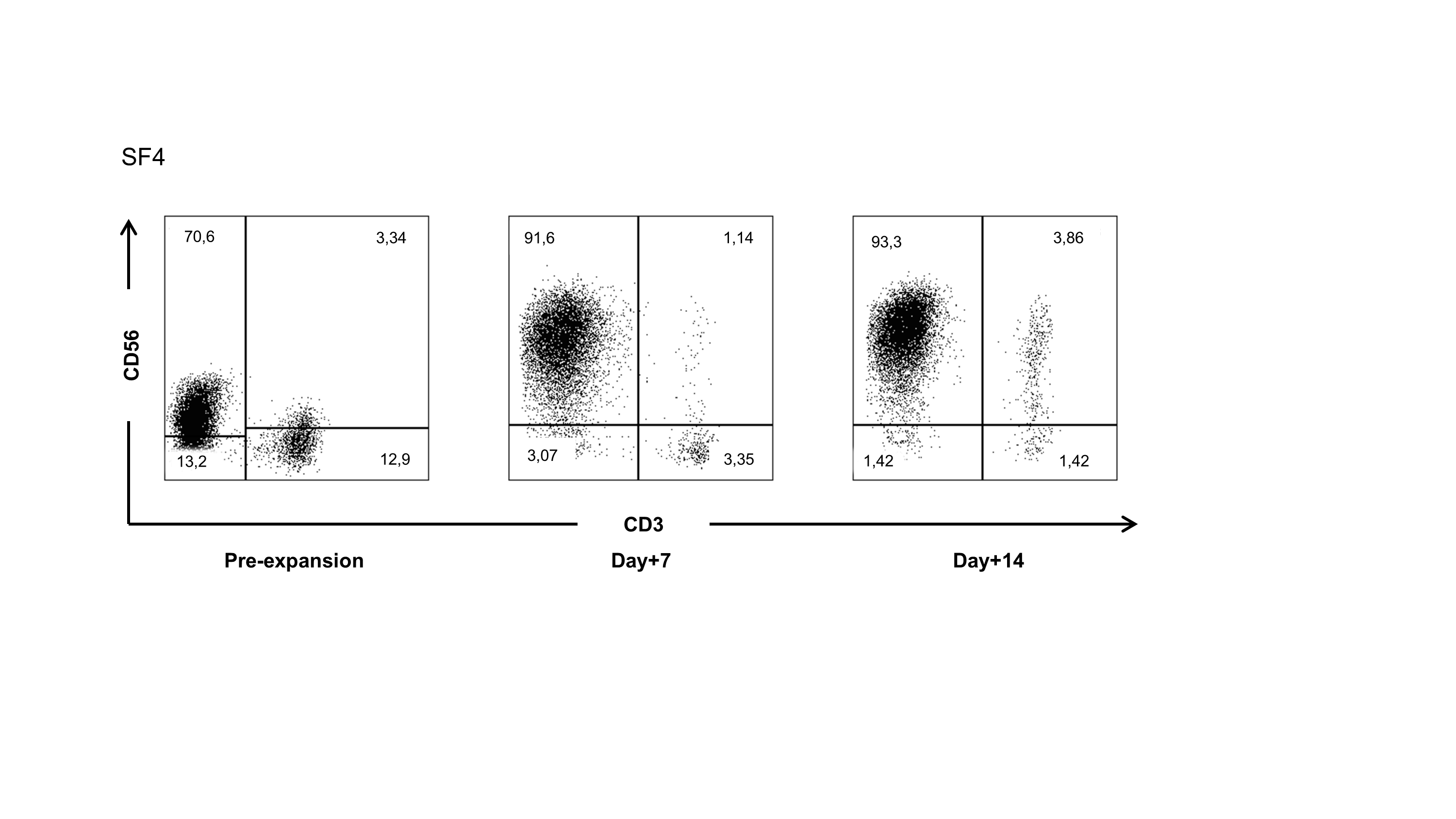

Supplement: Supplementary file 1 [file cancers-13-00577-s001.zip › SF4.tif]
