# Supplementary material for: Optimizing the Procedure to Manufacture Clinical-Grade NK Cells for Adoptive Immunotherapy
Source: Cancers (Basel). 2021 Feb 2;13(3):577. doi: 10.3390/cancers13030577 (PMC7867223; doi:10.3390/cancers13030577)
Supplement: Supplementary file 1 [file cancers-13-00577-s001.zip › ST2.doc]

**Supplementary Table 2.** Top 100 overexpressed and 100 underexpressed genes in stimulated NK cells when compared with unstimulated NK cells, ranked by fold change.

| **Overexpressed genes in stimulated NK cells** | | | **Underexpressed genes in stimulated NK cells** | | |
| --- | --- | --- | --- | --- | --- |
| **Genes** | **logFC** | **FDR** | **Genes** | **logFC** | **FDR** |
| IL17RB | 13,4 | 9,98E-73 | TNNT2 | 10,1 | 9,87E-22 |
| IL5 | 13,3 | 3,65E-69 | PMEPA1 | 9,5 | 4,34E-101 |
| PTGIS | 11,9 | 1,93E-28 | CD34 | 9,5 | 2,20E-13 |
| KRT7 | 11,8 | 6,71E-26 | DKK3 | 9,2 | 2,66E-10 |
| LINC00599 | 11,6 | 3,60E-84 | LRRC2 | 9,1 | 5,96E-10 |
| MND1 | 11,2 | 1,66E-21 | CCL24 | 9,0 | 1,23E-57 |
| TRBV10-2_1 | 10,9 | 2,41E-20 | COL13A1 | 9,0 | 5,67E-28 |
| TRBV10-2 | 10,9 | 2,53E-20 | CCL24_1 | 8,9 | 4,46E-53 |
| EPX | 10,9 | 5,71E-90 | RNF150 | 8,8 | 2,40E-08 |
| PRG3 | 10,8 | 9,54E-20 | GAB4 | 8,8 | 5,26E-08 |
| CDH17 | 10,6 | 2,50E-19 | PADI6 | 8,8 | 4,11E-08 |
| TRAV1-2 | 10,4 | 2,03E-18 | NCCRP1 | 8,7 | 1,31E-67 |
| IL4 | 10,0 | 9,65E-17 | F7 | 8,5 | 2,51E-32 |
| ALDH1A3 | 9,8 | 1,45E-15 | SPON2 | 8,3 | 4,52E-276 |
| LINC01132 | 9,8 | 1,69E-15 | PDGFB | 8,3 | 2,78E-06 |
| PTPN3 | 9,6 | 3,51E-14 | FPR3 | 8,3 | 3,16E-06 |
| RIBC2 | 9,4 | 3,78E-13 | SLC6A12 | 8,2 | 4,62E-06 |
| ENPEP | 9,4 | 7,30E-13 | PODN | 8,2 | 8,44E-62 |
| TRAV17 | 9,3 | 4,70E-12 | DGKK_1 | 8,2 | 4,44E-14 |
| CSF2 | 9,2 | 1,14E-49 | NKILA | 8,2 | 7,08E-06 |
| DPP4-DT | 9,2 | 1,51E-11 | DGKK | 8,1 | 1,89E-13 |
| IGHE | 9,2 | 2,37E-11 | TIFAB | 8,1 | 7,82E-06 |
| LOC105378047 | 9,2 | 4,57E-11 | LAD1 | 8,1 | 2,85E-66 |
| SRPX | 9,1 | 9,30E-11 | KRT72 | 8,1 | 6,93E-62 |
| LINC00892 | 9,0 | 8,66E-80 | LINC00487 | 8,0 | 2,10E-05 |
| IGHE_1 | 9,0 | 5,16E-10 | LPL | 8,0 | 1,74E-05 |
| TYMSOS | 9,0 | 9,32E-10 | SDC2 | 7,9 | 1,12E-15 |
| CCL23 | 8,9 | 3,52E-09 | EYA4 | 7,9 | 2,77E-05 |
| SPOCK1 | 8,8 | 7,58E-09 | OCA2 | 7,9 | 3,27E-05 |
| MB | 8,8 | 8,41E-09 | CLDN24 | 7,9 | 3,19E-05 |
| PCGF2 | 8,7 | 5,08E-08 | RBAKDN | 7,9 | 3,19E-05 |
| TRAV26-2 | 8,7 | 6,27E-08 | CDHR3 | 7,7 | 1,72E-24 |
| IL13 | 8,6 | 6,99E-66 | CMKLR1 | 7,7 | 6,26E-110 |
| MACC1 | 8,6 | 2,29E-07 | CAVIN2 | 7,6 | 0,00010894 |
| MYLK2 | 8,5 | 4,92E-07 | CD300E | 7,5 | 0,00019377 |
| CREB3L3 | 8,5 | 7,64E-46 | FLT3 | 7,4 | 2,31E-34 |
| BLOC1S5-TXNDC5 | 8,5 | 8,29E-07 | TM4SF19 | 7,4 | 6,12E-31 |
| TRAV4 | 8,5 | 9,96E-07 | KLF3-AS1 | 7,4 | 1,88E-14 |
| TRBV20-1 | 8,4 | 2,00E-06 | MYCT1 | 7,4 | 0,0002838 |
| IL26 | 8,4 | 2,40E-06 | LCNL1 | 7,3 | 1,26E-52 |
| BIVM-ERCC5 | 8,4 | 2,46E-06 | C6orf223 | 7,3 | 3,86E-07 |
| DIAPH3 | 8,4 | 2,69E-26 | FRMPD3 | 7,3 | 1,32E-61 |
| DMRT1 | 8,3 | 3,75E-06 | CLEC5A | 7,3 | 0,00038257 |
| RNASE2 | 8,3 | 4,64E-65 | CLEC5A_1 | 7,3 | 0,00038257 |
| CEACAM1 | 8,2 | 5,41E-20 | IDO1 | 7,3 | 4,26E-62 |
| EGFL6 | 8,2 | 6,33E-20 | WFDC21P | 7,2 | 0,00044992 |
| TRBV4-1 | 8,2 | 1,25E-05 | A2M | 7,2 | 3,33E-24 |
| TEX48 | 8,2 | 2,31E-05 | CYP27A1 | 7,2 | 1,35E-48 |
| ITGA2 | 8,2 | 2,83E-23 | ASGR2 | 7,2 | 0,00055105 |
| TRBV4-1_1 | 8,1 | 2,63E-05 | IGLV1-44 | 7,1 | 0,00063356 |
| TRBV7-2 | 8,1 | 5,87E-05 | PCDH1 | 7,1 | 4,35E-53 |
| MAGI1 | 8,1 | 5,74E-05 | NTN4 | 7,1 | 6,06E-21 |
| GNG12 | 8,0 | 5,44E-05 | CRHBP | 7,1 | 2,82E-06 |
| TRBV27_1 | 8,0 | 6,15E-05 | GPC4 | 7,1 | 3,74E-06 |
| TRBV12-4 | 8,0 | 9,68E-05 | PF4 | 7,1 | 2,03E-07 |
| SHISA2 | 8,0 | 9,72E-05 | ZMYND15 | 7,0 | 4,46E-26 |
| TRAV14DV4 | 8,0 | 0,00010625 | KIF13A | 7,0 | 1,00E-58 |
| CCDC80 | 7,9 | 0,0001259 | FOXD4L3_1 | 6,9 | 4,62E-16 |
| IL1A | 7,9 | 0,00015623 | FOXD4L3 | 6,9 | 2,91E-15 |
| TRAV2 | 7,9 | 0,00019377 | FABP4 | 6,9 | 2,01E-05 |
| LINC01775 | 7,8 | 0,00029086 | MIR181A2HG | 6,9 | 1,35E-20 |
| PBK | 7,8 | 1,11E-26 | SGCD | 6,8 | 2,83E-25 |
| CCL1 | 7,7 | 1,22E-17 | BOK | 6,8 | 2,34E-54 |
| DDIT4L | 7,7 | 0,00046644 | CLEC10A | 6,8 | 3,43E-06 |
| LOC100287036 | 7,7 | 0,00051789 | ZNF366 | 6,8 | 6,34E-10 |
| TRAV8-2 | 7,6 | 0,00082517 | RSPO4 | 6,7 | 5,57E-05 |
| MCOLN3 | 7,6 | 0,00092766 | VIPR2 | 6,7 | 3,79E-55 |
| IGHG4 | 7,6 | 0,00097239 | PTGDS | 6,7 | 4,63E-207 |
| LRRN3 | 7,6 | 2,92E-23 | PTPRB | 6,7 | 8,06E-13 |
| EXO1 | 7,6 | 9,07E-27 | LOC440896 | 6,7 | 1,73E-12 |
| HOXB5 | 7,5 | 0,00128645 | RASAL1 | 6,7 | 3,36E-21 |
| LOC102724450 | 7,5 | 0,00138512 | LGALS2 | 6,7 | 0,00010148 |
| TRAV12-2 | 7,5 | 0,00152121 | EPHA1 | 6,6 | 3,55E-116 |
| IGHG4_1 | 7,5 | 0,00144602 | PDZD4 | 6,6 | 2,15E-54 |
| SPC25 | 7,5 | 8,16E-18 | ERG | 6,6 | 0,00011431 |
| ELFN1-AS1 | 7,4 | 0,00205965 | PDZD4_1 | 6,6 | 1,13E-53 |
| PITX1 | 7,4 | 0,0021984 | SEMA3C | 6,6 | 2,74E-06 |
| LOC439933 | 7,4 | 0,002566 | ARHGAP22 | 6,5 | 3,05E-12 |
| TRBV27 | 7,4 | 0,00271262 | DSC2 | 6,5 | 5,22E-08 |
| CTLA4 | 7,4 | 1,81E-30 | NKD1 | 6,4 | 1,81E-72 |
| TYMS | 7,4 | 4,35E-48 | PROM1 | 6,4 | 8,88E-05 |
| AICDA | 7,4 | 0,0029285 | SERPINF1 | 6,4 | 1,17E-17 |
| TRAV1-1 | 7,3 | 0,00316587 | ALDH1A2 | 6,3 | 0,00195978 |
| TRAV8-6 | 7,3 | 0,00373705 | CCDC68 | 6,3 | 0,00010758 |
| RAET1K | 7,3 | 0,00373434 | PSD3 | 6,3 | 2,80E-24 |
| SPRY4 | 7,3 | 0,00419581 | TPD52L1 | 6,3 | 0,00067268 |
| CA10 | 7,2 | 0,00470191 | NUAK2 | 6,3 | 1,34E-67 |
| IQGAP3 | 7,2 | 4,56E-63 | AMY2A | 6,2 | 2,51E-08 |
| TCN1 | 7,2 | 0,00553111 | ZNF727 | 6,2 | 2,71E-07 |
| TRBV11-3 | 7,2 | 0,00601612 | TBC1D9 | 6,2 | 9,12E-23 |
| LRG1 | 7,1 | 0,00661883 | RAB31 | 6,2 | 1,41E-48 |
| LINC02694 | 7,1 | 1,95E-16 | MOCS1 | 6,2 | 2,62E-05 |
| E2F7 | 7,1 | 1,64E-29 | EFHC2 | 6,1 | 6,84E-24 |
| INSYN2A | 7,1 | 0,00835072 | PPBP | 6,1 | 0,00136265 |
| STC2 | 7,1 | 0,00854531 | TFPI | 6,1 | 5,61E-09 |
| MYBL2 | 7,0 | 1,30E-56 | FAM20A | 6,1 | 1,09E-06 |
| TRAV13-1 | 7,0 | 0,00941253 | ARNT2 | 6,1 | 3,78E-08 |
| TRBV9_1 | 7,0 | 0,00979793 | HSD17B3 | 6,1 | 1,21E-08 |
| KCNA2 | 7,0 | 1,01E-17 | ETNK2 | 6,1 | 9,70E-17 |
| CDC25A | 7,0 | 1,37E-28 | TRPV3 | 6,1 | 1,18E-37 |
